# Endocannabinoids enhance hK_V_7.1/KCNE1 channel function and shorten the cardiac action potential and QT interval

**DOI:** 10.1016/j.ebiom.2023.104459

**Published:** 2023-02-14

**Authors:** Irene Hiniesto-Iñigo, Laura M. Castro-Gonzalez, Valentina Corradi, Mark A. Skarsfeldt, Samira Yazdi, Siri Lundholm, Johan Nikesjö, Sergei Yu Noskov, Bo Hjorth Bentzen, D. Peter Tieleman, Sara I. Liin

**Affiliations:** a Department of Biomedical and Clinical Sciences, Linköping University, Linköping, Sweden; b Centre for Molecular Simulation and Department of Biological Sciences, University of Calgary, Calgary, AB, Canada; c Department of Biomedical Sciences, University of Copenhagen, Copenhagen, Denmark

**Keywords:** Arrhythmia, Electrophysiology, KCNQ1, Kv7, Long QT Syndrome, Molecular dynamics

## Abstract

**Background:**

Genotype-positive patients who suffer from the cardiac channelopathy Long QT Syndrome (LQTS) may display a spectrum of clinical phenotypes, with often unknown causes. Therefore, there is a need to identify factors influencing disease severity to move towards an individualized clinical management of LQTS. One possible factor influencing the disease phenotype is the endocannabinoid system, which has emerged as a modulator of cardiovascular function. In this study, we aim to elucidate whether endocannabinoids target the cardiac voltage-gated potassium channel K_V_7.1/KCNE1, which is the most frequently mutated ion channel in LQTS.

**Methods:**

We used two-electrode voltage clamp, molecular dynamics simulations and the E4031 drug-induced LQT2 model of *ex-vivo* guinea pig hearts.

**Findings:**

We found a set of endocannabinoids that facilitate channel activation, seen as a shifted voltage-dependence of channel opening and increased overall current amplitude and conductance. We propose that negatively charged endocannabinoids interact with known lipid binding sites at positively charged amino acids on the channel, providing structural insights into why only specific endocannabinoids modulate K_V_7.1/KCNE1. Using the endocannabinoid ARA-S as a prototype, we show that the effect is not dependent on the KCNE1 subunit or the phosphorylation state of the channel. In guinea pig hearts, ARA-S was found to reverse the E4031-prolonged action potential duration and QT interval.

**Interpretation:**

We consider the endocannabinoids as an interesting class of hK_V_7.1/KCNE1 channel modulators with putative protective effects in LQTS contexts.

**Funding:**

10.13039/100000190ERC (No. 850622), 10.13039/501100000024Canadian Institutes of Health Research, 10.13039/501100001804Canada Research Chairs and 10.13039/100013020Compute Canada, Swedish National Infrastructure for Computing.

**Research in context**

**Evidence before this study**

The heart rhythm is maintained by the electrical activity of several ion channels and transporters. Mutations in different ion channels have been linked to the arrhythmia Long QT Syndrome (LQTS), in which the K_V_7.1/KCNE1 channel is the most frequently mutated channel. However, there is individual variability in the disease severity of LQTS, which may be caused by endogenous factors that modulate ion channels.

**Added value of this study**

We find that endocannabinoids are potent activators of the K_V_7.1/KCNE1 channel and provide mechanistic insights into endocannabinoid binding sites and mechanisms of action. Moreover, we find that endocannabinoids restore a physiological QT interval in a guinea pig model of LQTS.

**Implications of all the available evidence**

Combined, these findings show that endocannabinoids may act as disease modifiers in LQTS.

## Introduction

Congenital Long QT Syndrome (LQTS) is an inherited cardiac channelopathy characterised by a delayed ventricular repolarization.^1^ Clinically, LQTS is detected as a prolonged QT interval on the electrocardiogram, and a predisposition for LQTS-triggered cardiac events including syncope, ventricular fibrillation, and in the worst case sudden cardiac death, often during times of emotional or physical stress.^2^ Over the past decades, 15 distinct LQTS-susceptibility genes, some encoding pore-forming alpha or auxiliary subunits of key cardiac ion channels, have been identified.^3^ As knowledge of the genetic basis of LQTS continues to expand, an overall goal is to develop individualized anti-arrhythmic risk assessment and therapy.^4^ However, the presence of a genetic mutation alone cannot always guide clinical management, as genotype-positive family members may display a spectrum of clinical phenotypes.^5^^,^^6^ As such, the interplay between genotype and phenotype in LQTS is likely far more complex than previously envisioned.^7^^,^^8^ The identification of factors influencing disease severity would be one important step towards a more precise risk stratification and individualized clinical management of LQTS.

**Fig. 1:**
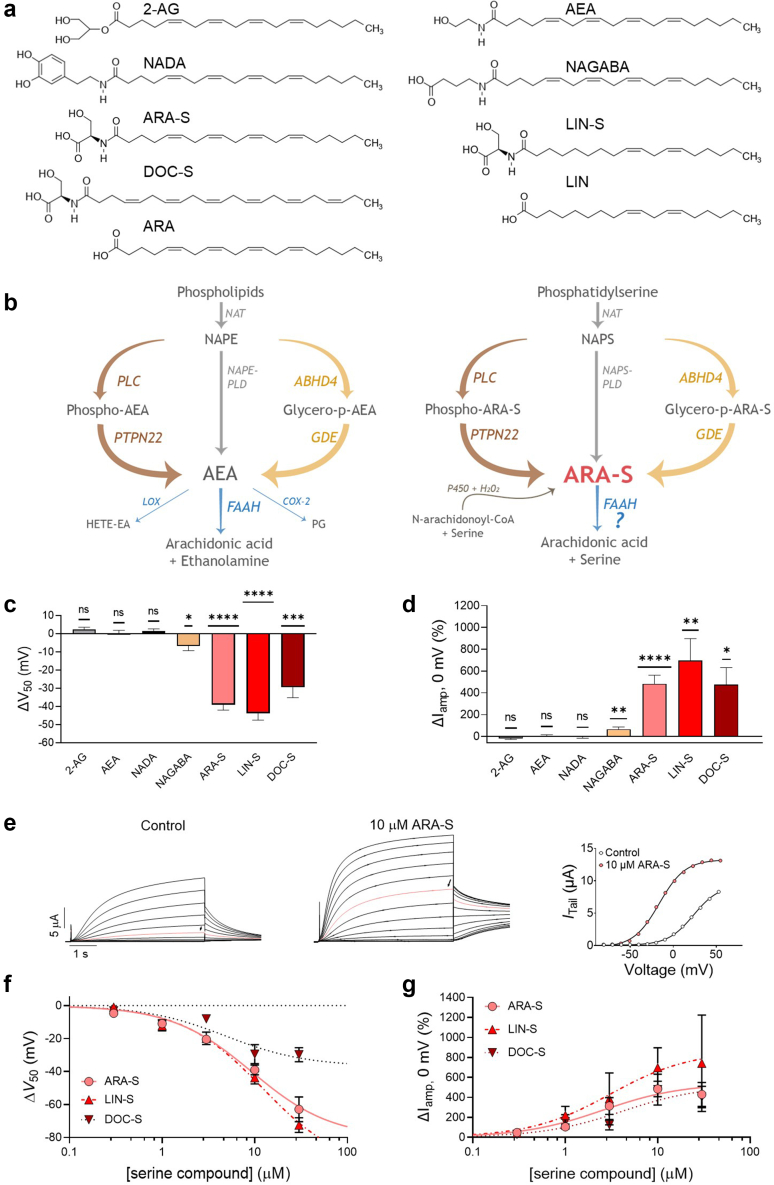
**Specific endocannabinoids activate the hK**_**V**_**7.1/KCNE1 channel.** Effect of indicated endocannabinoids on hK_V_7.1/KCNE1 expressed in *Xenopus* oocytes and studied with the two-electrode voltage clamp technique. a) Molecular structure of 2-AG, NADA, AEA, NAGABA, ARA-S, LIN-S and DOC-S. Structures of the PUFAs arachidonic acid (ARA) and linoleic acid (LIN) are included for comparison. b) Side-by-side comparison of the metabolic pathways of AEA (left) and the hypothetical metabolic pathway of ARA-S (right). See text for details. Abbreviations: AEA, *N-*arachidonoylethanolamide (anandamide); NAT, *N-*acyltransferase; NAPE, *N-*acyl-phosphatidylethanolamide; NAPE-PLD, NAPE-specific phospholipase D; NAPS, *N-*arachidonoylphosphatidylserine; NAPS-PLD; NAPS-specific phospholipase D; ABHD4, α/β-hydrolase domain 4; GDE, glycerolphosphodiesterase; PLC, phospholipase C; PTPN22, non-receptor protein tyrosine phosphatase 22; FAAH, fatty acid amide hydrolase; COX-2, cyclooxygenase-2; LOX, lipoxygenase; PG-EA, prostaglandin-ethanolamide; HETE-EA, hydroxyeicosatetraenoyl-ethanolamide; P450, cytochrome P450 monooxygenase. c and d) Mean shift in (c) ΔV_50_ and (d) ΔI_amp_ at 0 mV induced by 10 μM of indicated endocannabinoids on hK_V_7.1/KCNE1. e) Representative traces of hK_V_7.1/KCNE1 currents under control conditions and in the presence of 10 μM ARA-S (red traces indicate an activating voltage step to 0 mV) and corresponding G(V) curve. For this specific cell: V_50;ctrl_ = +23.6 mV, I_tailmax;ctrl_ = 9.3 μA, V_50;ARA-S_ = −16.2 mV, I_tailmax;ARA-S_ = 13.2 μA. Currents were generated in steps from −80 to +50 mV in 10 mV steps, followed by a tail voltage of −20 mV. The holding voltage was −80 mV. f) Concentration-response relation for ΔV_50_ effect ARA-S, LIN-S and DOC-S. Best fit: ARA-S: EC_50_ = 9 μM, ΔV_50, max_ = −80.2 mV; LIN-S: EC_50_ = 12 μM, ΔV_50, max_ = −99.5 mV; DOC-S: EC_50_ = 5 μM, ΔV_50, max_ = −36.9 mV. g) Concentration-response relation for ΔI_amp_ at 0 mV effect of ARA-S, LIN-S and DOC-S. Best fit: ARA-S: EC_50_ = 2.5 μM, ΔI_amp,max_ = +540%; LIN-S: EC_50_ = 3 μM, ΔI_amp,max_ = +865%; DOC-S: EC_50_ = 4 μM, ΔI_amp,max_ = +522%. Statistics indicate one-sample *t* test compared to a hypothetical value of 0 (i.e. no change in V_50_ or current amplitude). ∗ denotes P < 0.05, ∗∗ denotes P < 0.01, ∗∗∗ denotes P < 0.001, ∗∗∗∗ denotes P < 0.0001. P > 0.05 (ns). Data shown as mean ± SEM; n = 7–12.

The endocannabinoid system has emerged as a putative modulator of cardiovascular function. This system includes the two best known lipid-based endocannabinoids 2-arachidonoyl glycerol (2-AG) and anandamide (N-arachidonoyl ethanolamine, AEA)^9^ (structures in Fig. 1a) and other structurally related endocannabinoid-like lipids (commonly also referred to as endovanilloids or N-acyl amides).^10^^,^^11^ The number of identified endocannabinoid-like lipids has rapidly grown in recent years. Several compounds, primarily with arachidonic acid tails (one of the most common acyl tails) and different head groups such as serine or dopamine (examples shown in Fig. 1a), have been detected in human plasma.^12^ For simplicity, we will refer to all of these as endocannabinoids. Although all these compounds follow the general metabolic pathways of 2-AG or AEA, the precise metabolic pathway of each N-acyl amide is not fully understood. Fig. 1b shows a side-by-side comparison of the known metabolic pathways of AEA^13^ and the hypothetical synthesis and degradation pathways of N-arachidonoyl-L-serine (ARA-S),^14^ which has an arachidonic acid tail and a serine head group. The synthesis of ARA-S may start with the conversion of phosphatidylserine (PS) to N-arachidonoylphosphatidylserine (NAPS) by an acyltransferase.^15^^,^^16^ Then, ARA-S is produced following one of three possible pathways: phospholipase D (PLD) hydrolysis,^17^ phospholipase C (PLC) hydrolysis followed by dephosphorylation by the PTPN22 phosphatase,^15^ or by deacylation by the ABH4 hydrolase, followed by N-acylphosphatidyl (PDE) hydrolysis. In addition, there is a fourth possible pathway, in which P450 catalyses the reaction of N-arachidonoyl CoA and serine with H_2_0_2_ to synthesize ARA-S.^18^ Regarding the degradation of ARA-S, it is proposed that the fatty acid amide hydrolase (FAAH) is involved in the breakdown of ARA-S into arachidonic acid and serine. In agreement with this, elevated levels of ARA-S have been found in FAAH knock out mice models and when pharmacologically inhibiting FAAH.^19^^,^^20^ However, the breakdown of N-acyl amides by FAAH seems to be tissue-dependent, with neglectable changes in the levels of N-acyl amides in the heart upon FAAH inhibition.^21^^,^^22^ Although the exact metabolic pathways and local abundance of many endocannabinoids under physiological and pathological conditions in the heart remain largely unknown,^11^ it is likely that their abundance increases in various cardiovascular disorders (e.g. different forms of shock, cardiomyopathies, atherosclerosis).^23^ In addition to canonical endocannabinoid signalling through cannabinoid receptors,^9^ physiological and pathological effects are mediated through noncanonical targets such as ion channels.^23^ For instance, 2-AG and AEA prolong atrial action potential duration (APD) by inhibiting the cardiac potassium channels K_V_4.3 and K_V_1.5,^24^^,^^25^ modulate cardiac muscle contractility through the inhibition of Na_V_ and L-type Ca_V_ channels in ventricular myocytes,^26^ and mediate vasodilatory effects through activation of TRPV1.^27^ ARA-S has been found in the cardiovascular system to exert vasodilatory effects^28^ and in neuronal tissue^29^^,^^30^ to tune neuronal excitability.^31^ Some of these effects are likely to involve the activation of the hK_V_7.2/7.3 channel,^31^^,^^32^ the BK channel,^33^ and the N-type Ca_V_ channel.^34^

However, whether endocannabinoids also target the cardiac voltage-gated potassium channel K_V_7.1/KCNE1, which is the most commonly mutated channel in LQTS, remains unstudied. K_V_7.1/KCNE1 generates the slowly activating component of the delayed rectifier K^+^ current (I_Ks_) in cardiomyocytes, which contributes to cardiomyocyte repolarization and is essential for the physiological shortening of the APD and QT interval triggered by sympathetic stimulation of the heart.^35^ The channel complex is composed of K_V_7.1 and up to four auxiliary KCNE1 subunits.^36^ K_V_7.1 has the general structure of voltage-gated K^+^ channels formed through tetramerization of channel subunits. Each subunit has 6 transmembrane segments (S1 to S6), of which S1-S4 form the voltage-sensing domains (VSD) and segments S5-S6 form a common pore domain (PD).^37^ Upon depolarization, the voltage sensor S4 moves upwards and, because it is electromechanically coupled to the PD, the ion permeation pathway opens allowing for outward repolarizing K^+^ currents.^38^^,^^39^ KCNE1 has one transmembrane segment.^40^ Based on the preference of K_V_7.1/KCNE1 for interacting with different types of lipids, including the phospholipid phosphatidylinositol 4,5-bisphosphate (PIP_2_)^37^^,^^41^ and polyunsaturated fatty acids (PUFAs),^42^, ^43^, ^44^, ^45^, ^46^, ^47^ we hypothesized that K_V_7.1/KCNE1 may be a non-canonical cardiac target of endocannabinoids.

Using the two-electrode voltage clamp technique on *Xenopus* oocytes expressing the human K_V_7.1/KCNE1 (hK_V_7.1/KCNE1) channel, we found that negatively charged endocannabinoids, like ARA-S, target hK_V_7.1/KCNE1 and facilitate channel activation, seen as a shifted voltage-dependence of channel opening and increased overall current amplitude and conductance. Computer simulations together with experiments suggest that endocannabinoids interact with known PUFA binding sites at positively charged amino acids on the channel and provide mechanistic insights into why only negatively charged endocannabinoids are effective. The effect does not depend on the KCNE1 subunit or the phosphorylation state of the channel. In guinea pig hearts, ARA-S reverses drug-induced prolongation of the APD and QT interval. Hence, we consider the endocannabinoids as an interesting class of hK_V_7.1/KCNE1 channel modulators with putative protective effects in LQTS contexts.

## Methods

Detailed materials and methods are described in the SI Appendix.

### Ethics

*Xenopus laevis* experiments were approved by the Linköping Animal Care and Use Committee (Permit #1941) and conform to national and international guidelines. Guinea pig experiments were performed at the Department of Biomedical Sciences, University of Copenhagen, Denmark and done according to the Danish guidelines for animal experiments under license 2017-15-0201-01296.

### Two-electrode voltage clamp experiments on *Xenopus* oocytes

*Xenopus laevis* oocytes were isolated and maintained as previously described.^32^ Oocytes were injected with RNA for human K_V_7.1, KCNE1 and/or Ci-VSP as indicated in each results section. Two-electrode voltage clamp experiments were performed using a Dagan CA-1B amplifier (Dagan, MN, USA) or an AxoClamp 900 A amplifier (Molecular Devices, CA, USA). All endocannabinoids were bought from Cayman Chemicals (MI, USA), except ACEA which was bought from Tocris Bioscience (Bristol, UK). DOC-S, LIN-S, ARA-D-S were synthesized in house as described previously.^32^

To quantify the voltage dependence of channel opening, tail currents were measured shortly after stepping to the tail voltage and plotted against the preceding activation voltage. A Boltzmann function was fitted to the data to generate the conductance versus voltage G(V) curve:

(1) $$G\left(V\right)=G_{\mathrm{MIN}}+\left(G_{\mathrm{MAX}}-G_{\mathrm{MIN}}\right)/\left\{1+\exp\left[\frac{V_{50}-V}{s}\right]\right\}$$

where G_MIN_ is the minimum conductance, G_MAX_ the maximum conductance, V_50_ the midpoint (i.e., the voltage at which the conductance is half the maximum conductance determined from the fit), and s the slope of the curve.

To plot the concentration dependence of the compound-induced effect as a function of the compound concentration, the following concentration–response curve was fitted to the data:

(2) $$\Delta Effect=\Delta {Effect}_{\mathrm{MAX}}/\left[1+{\left(\frac{EC_{50}}{C}\right)}^{H}\right]$$

where ΔEffect_MAX_ is the maximal shift in V_50_, the maximal change in current amplitude or the maximal change in G_MAX_, EC_50_ the concentration needed to cause 50% of the maximal effect, and H the Hill coefficient (set to 1).

### SILCS and molecular dynamics simulations

We used the Site-Identification by Ligand Competitive Saturation (SILCS) software^48^ to identify putative binding sites for LIN, LIN-S, ARA and ARA-S in K_V_7.1. As a starting structure, we used the *Xenopus laevis* K_V_7.1 structure, solved by cryo-EM at 3.7 Å (PDB: 5VMS).^49^ In describing the results, the human K_V_7.1 numbering is used. Molecular dynamics simulations were performed using the transmembrane domain (residues 104-358) of the Cryo-EM structure of human K_V_7.1, solved at 3.1 Å.^37^ Further details for SILCS, MD simulations and analyses are provided in the Supporting information.

### Isolated heart experiments

The Langendorff-perfused heart experiments were performed as described in Liin et al.,^47^ 16 adult female Dunkin Hartley guinea pigs (300–440 g) from Charles River, France were used. The electrical activity was measured using volume conducted electrocardiogram (ECGs) and by placing epicardiac monophasic action potential (MAP) electrodes on RV and LV (Hugo Sachs Elektronik-Harvard Apparatus GmbH, March-Hugstetten, Germany) to collect electrophysiological parameters when perfusing 0.03 μM E4031 and 1 μM, 3 μM and 10 μM ARA-S, or equivalent ethanol for time matched control.

### Statistical analysis

Average values are expressed as mean ± SEM. Statistics were calculated using one-sample *t* test (to compare with a hypothetical value of 0), Student's *t* test to compare between two groups or one-way ANOVA followed by Tukey's multiple comparisons test to compare multiple groups. For the isolated heart experiments, two-way ANOVA with Dunnett's multiple comparisons test was used. P < 0.05 was considered statistically significant. All statistical analyses were carried out in GraphPad Prism 9.

### Role of the funding source

The funding sources had no involvement in the study design, the collection, analysis, and interpretation of data, and in the writing of the manuscript.

## Results

### Specific endocannabinoids facilitate activation of the hK_V_7.1/KCNE1 channel

To study whether endocannabinoids target the hK_V_7.1/KCNE1 channel, we tested the effect of 10 μM of the endocannabinoids 2-AG, AEA, NADA, NAGABA, ARA-S, LIN-S and DOC-S (structures in Fig. 1a) on human K_V_7.1 and KCNE1 co-expressed in *Xenopus* oocytes. Since previous work has shown that a polyunsaturated tail is required for related lipid compounds to interact with hK_V_7.1,^47^ we focused our efforts on endocannabinoids with polyunsaturated tails. We found that 10 μM of the most abundant endocannabinoids—2-AG, AEA and NADA—which have an arachidonic acid tail and either glycerol, ethanolamide, or dopamine head group (Fig. 1a), had no clear effect on the hK_V_7.1/KCNE1 channel (Figs. 1c, S1a–c, note that statistics are reported in figures and tables throughout the manuscript). In response to endocannabinoid application, V_50_ and G_MAX_ remained within 0–3 mV and 0–17%, respectively (Figs. 1c, S1a, Table 1). To confirm that the lack of effect seen for 2-AG, AEA and NADA was not due to stability issues of these compounds, we tested the uncharged arachidonyl-2′-chloroethylamide (ACEA) (structure in Fig. S1b),^50^ a more stable AEA analogue. Similar to the other uncharged compounds, ACEA had no effect on V_50_ (ΔV_50_ = +3.0 ± 1.9 mV, P > 0.05, n = 6, one-sample *t* test). For G_MAX_, only one cell out of 6 responded with a noticeable G_MAX_ increase, rendering a non-significant G_MAX_ change of +65 ± 47% (P > 0.05, n = 6, one-sample *t* test or non-parametric Wilcoxon's test) (Table 1). Hence, the ACEA experiments verify that the lack of effect of the uncharged endocannabinoids is not because of stability issues. 10 μM of NAGABA, which has an arachidonic acid tail and a GABA head group (Fig. 1a), moderately facilitated activation of the hK_V_7.1/KCNE1 channel by shifting V_50_ by −6.7 ± 2.6 mV and increasing G_MAX_ by 23 ± 4% (Figs. 1c, S1a, Table 1). 10 μM of the endocannabinoids ARA-S, LIN-S and DOC-S, which have diverse tails and a serine head group, prominently facilitated activation of the hK_V_7.1/KCNE1 channel by shifting V_50_ by up to −43.8 ± 3.8 mV and increasing G_MAX_ by up to 96 ± 26% (Figs. 1c, S1a, Table 1). We also quantified the effect of each compound on the steady-state current at 0 mV, which is a voltage relevant for the plateau phase of the ventricular action potential.^51^ In agreement with the V_50_ and G_MAX_ effects, 10 μM of 2-AG, AEA, and NADA had no effect on current amplitude at 0 mV and NAGABA moderately increased the current amplitude by 66 ± 20% (Fig. 1d, Table 1). In contrast, ARA-S, LIN-S and DOC-S prominently increased the current amplitude at 0 mV by 477 ± 155%–698 ± 199% (Fig. 1d, Table 1). In summary, the endocannabinoids tested show a range of effects on hK_V_7.1/KCNE1, from no effect (representative example of 2-AG in Fig. S1c) to clear activating effects: Facilitating channel opening at more negative voltages and inducing larger overall current amplitude (representative example of ARA-S in Fig. 1e). The serine-based endocannabinoids showed concentration-dependent effects on hK_V_7.1/KCNE1 (Fig. 1f and g), with the magnitude of effects in overall agreement for the compounds. However, ARA-S and LIN-S appeared to induce larger estimated maximal effects on V_50_ than DOC-S did (Fig. 1f, please refer to figure legends for best fit details for Fig. 1f and g). The effect on G_MAX_ was less robust and did not always show a simple concentration dependence, mainly because of reduced G_MAX_ effects at the highest concentrations (Fig. S1d). Therefore, throughout the remainder of the work we will primarily focus on the more robust effects on V_50_ and current amplitude at 0 mV.

**Table 1:** Summary of effect induced by 10 μM of indicated compound on hK_V_7.1/KCNE1.

Compound	Δ*V*_50_ (mV)	P	Δ*I*_*amp*_, _*0 mV*_ (%)	P	Δ*G*_*MAX*_ (%)	P	n
2-AG	+2.4 ± 1.2	0.1	−18 ± 10	>0.1	0 ± 6	>0.5	7
AEA	+0.1 ± 1.7	>0.5	+5 ± 12	>0.5	+16 ± 6	<0.01	7
NADA	+1.5 ± 1.2	>0.1	−4 ± 13	>0.5	+5 ± 8	>0.5	10
ACEA	+3.0 ± 1.9	>0.1	+33 ± 52	>0.5	+65 ± 48	>0.1	6
NAGABA	−6.7 ± 2.6	<0.05	+66 ± 20	<0.01	+23 ± 4	<0.0001	12
ARA-S	−39.0 ± 3.0	<0.0001	+484 ± 78	<0.001	+57 ± 24	<0.05	12
LIN-S	−43.8 ± 3.8	<0.0001	+698 ± 199	<0.01	+96 ± 26	<0.01	10∗
DOC-S	−29.4 ± 5.8	<0.001	+477 ± 155	0.01	+34 ± 20	>0.1	9
ARA-Serinol	+1.1 ± 0.8	>0.5	+7 ± 23	>0.1	+2 ± 2	0.5	8
ARA-D-S	−29.0 ± 2.3	<0.0001	+587 ± 119	<0.01	+55 ± 14	<0.05	6
NALA	−22.2 ± 1.9	<0.0001	+646 ± 174	<0.01	+77 ± 13	<0.01	6
NAGly	−25.5 ± 1.9	<0.0001	+620 ± 202	<0.01	+73 ± 8	<0.001	6
AA-5HT	−1.7 ± 1.5	>0.1	+60 ± 29	>0.05	+33 ± 7	<0.01	6

Data shown as mean ± SEM. Effects on indicated parameters were determined from Boltzmann fits, as described in the Methods section. Statistics denote one-sample *t* test compared to a hypothetical value of 0 (i.e., no change in indicated parameter). n indicates the number of recordings (∗n = 10 for LIN-S except for Δ*I*_*amp*, *0 mV*_ for which n = 9).

### SILCS and molecular dynamics simulations retrieve known lipid binding sites and specific interactions with K_V_7.1

**Fig. 2:**
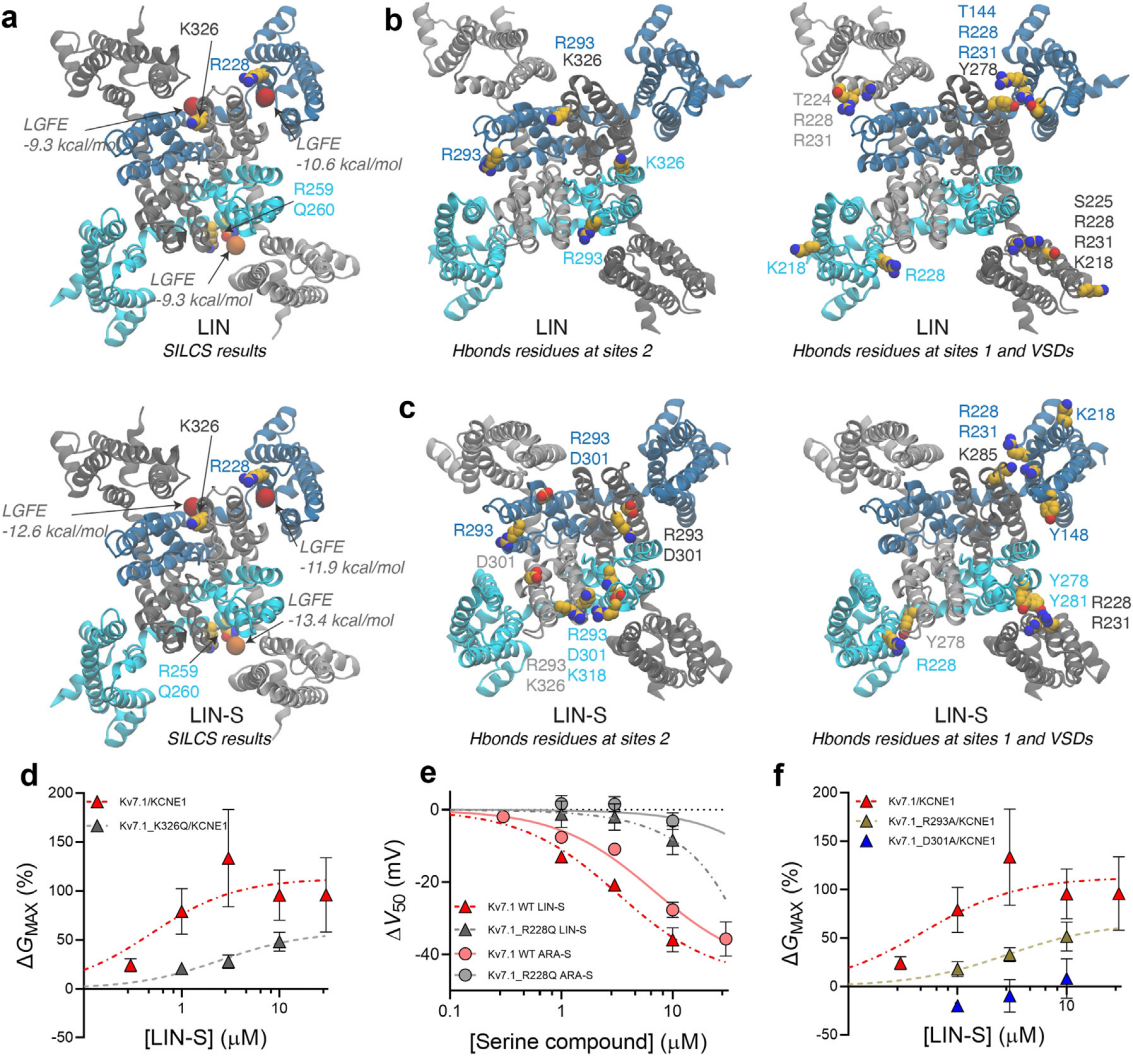
**LIN and LIN-S regions of interactions with K**_**V**_**7.1.** Computational analyses of endocannabinoid interactions with hK_V_7.1, and experimental assessment of the impact of introducing indicated mutations on the endocannabinoid effect on hK_V_7.1 or hK_V_7.1/KCNE1. a) SILCS results highlighting the centroid of regions of interactions for the PUFA LIN (upper panel) and endocannabinoid LIN-S (*lower panel*). The centroids are shown as red and orange spheres for regions in the upper and lower leaflet, respectively. The SILCS score for each region is reported, and known PUFAs binding residues^45^ at sites 1 (R228) and 2 (K326) and additional residues in the lower leaflet are shown as spheres, as a reference. b and c) Residues in the upper leaflet engaging in hydrogen bonds with (b) LIN and (c) LIN-S at sites 2, sites 1 and additional regions in the VSDs. The residues are labelled according to the color of the corresponding monomer, cyan for chain A, light gray for chain B, blue for chain C and gray for chain D. The total count of the hydrogen bonds was scaled by the number of frames, and only residues forming a minimum of 0.5 hydrogen bonds per frame are shown. In b and c, the protein represents the last frame of the simulation. d) Concentration-response relation for ΔG_MAX_ of LIN-S in hK_V_7.1/KCNE1 WT and hK_V_7.1_K326Q/KCNE1. Best fit for hK_V_7.1_K326Q/KCNE1: EC_50_ = 2.5 μM, ΔG_MAX, max_ = 58%. Data shown as mean ± SEM; n = 4–10. Note that the 30 μM concentration was excluded from the fit. Note that hK_V_7.1_K326Q does not generate measurable currents without KCNE1 co-expression.^46^ e) Concentration-response relation for ΔV_50_ of LIN-S and ARA-S in hK_V_7.1 WT and hK_V_7.1_R228Q. Best fit = ambiguous. Data shown as mean ± SEM; n = 4–9. The effect of 10 μM of ARA-S is comparable to the effect previously reported.^32^ f) Concentration-response relation for ΔG_MAX_ of LIN-S in hK_V_7.1/KCNE1 WT, hK_V_7.1_R293A/KCNE1 and hK_V_7.1_D301A/KCNE1. Best fit for hK_V_7.1_R293A/KCNE1: EC_50_ = 2.8 μM, ΔG_MAX, max_ = 65%. Best fit for hK_V_7.1_D301A/KCNE1 = ambiguous. Data shown as mean ± SEM; n = 4–10.

Previous work has shown that PUFAs and several analogues facilitate activation of hK_V_7.1/KCNE1, seen as a shifted V_50_ and increased current amplitude, through direct binding to the K_V_7.1 channel.^44^^,^^45^^,^^47^ Recently, we combined molecular dynamics (MD) simulations with electrophysiology experiments to characterize the interaction between linoleic acid (LIN) and K_V_7.1^45^ and identified two functional LIN sites on the extracellular end of K_V_7.1: One site (referred to as site 1) next to the S4 gating charges (R228) in the VSD, and the other (referred to as site 2) next to K326 in S6 in the PD.^45^ To determine if the chemically related lipid-based endocannabinoids studied in this work utilize the same overall binding sites as LIN, we performed Site Identification by Ligand Competitive Saturation (SILCS) calculations and MD simulations. SILCS generates functional group free energy maps (FragMaps) for a given protein, and uses them to identify possible regions of interactions of the ligands of choice, ranked based on the Ligand Grid Free Energy (LGFE) score (Fig. S2).^48^^,^^52^, ^53^, ^54^ Here, we applied SILCS to K_V_7.1 and a library of four ligands, i.e. arachidonic acid (ARA), LIN, ARA-S and LIN-S (structures in Fig. 1a), and we analysed the top three interaction regions for each ligand (Figs. 2a and S3). For all ligands, in the upper leaflet, SILCS identified the centroid of these regions in proximity of the positively charged residues previously reported^45^ for site 1 (R228) and site 2 (K326) (Figs. 2a and S3). Near the intracellular end of the transmembrane domains, between the linker S4-S5 and S5, an additional interacting region is centred close to R259 and Q260 (Figs. 2 and S3). These results not only confirmed the known binding sites (site 1 and site 2)^45^ for PUFAs with the carboxylic head group (LIN and ARA), but also suggested that the endocannabinoids LIN-S and ARA-S can interact with K_V_7.1 at these sites.

We explored further the interactions between K_V_7.1 and compounds with a carboxylic or a serine head group with 5 μs-long atomistic MD simulations of K_V_7.1 embedded in a multi-component membrane, in the presence of LIN or LIN-S. We first calculated the number density for LIN and LIN-S in each leaflet, using the last 1 μs of the simulations (Fig. S4). For both ligands, in the upper leaflet, the density is found symmetrically distributed around the tetrameric channel near the previously reported site 2^45^ and in the nearby groove, lined primarily by the pore helix of one monomer and surrounding extracellular loops, by the S6 helix of a second monomer, and by the S1 helix of a third monomer (Fig. S4a). We then identified the protein residues at this region that formed hydrogen bonds with LIN and LIN-S (see SI Appendix Methods for details and Fig. S5). We found that LIN-S engaged with a higher number of residues compared to LIN (Figs. 2b and c and S5). In particular, from the monomer contributing to the interface with its pore helix, R293 and D301 are the residues that formed the highest number of hydrogen bonds with LIN-S. Interestingly, for a given site 2 and bound ligands, R293 from the neighbouring monomer can contribute to the interactions with LIN-S, indicating the flexibility of the loop carrying R293. K326, the positively charged residue previously reported for site 2,^45^ is also found among the residues interacting with LIN-S, although to a lesser degree when compared with R293 and D301. Residues from the S1 segment, e.g. residues at the positions 144–148, only occasionally formed hydrogen bonds. LIN, on the other hand, established the highest number of hydrogen bonds with R293, although in only two of the four sites 2 of the tetramer (Figs. 2b and c and S5). Similar to what we observed for LIN-S, R293 can engage with different sites 2. We also observed that K326 can interact with LIN (Figs. 2b and c and S5). Outside the previously described region around site 2, ligand density was detected around the VSDs and near site 1,^45^ located at the interface between the VSD of one monomer and the S5 helix of the neighbouring monomer, for both LIN and LIN-S, although more pronounced for LIN-S (Fig. S4). The hydrogen bond analysis identified primarily residues from the VSDs of different monomers as well as R228 and R231 (both residues from the previously described site 1)^45^ as common residues of interaction for both LIN-S and LIN (Figs. 2b and c, and S6). For site 1, LIN-S can engage with additional S5 residues, namely Y278, Y281, or K285 (Figs. 2b and c, and S6). In the case of LIN, on the other hand, for only one interface we retrieved Y278. In addition, LIN can intercalate among the extracellular ends of the VSD helices, projecting its head group towards R228, R231 and nearby residues.

In the lower leaflet, LIN-S density is particularly noticeable along the linker connecting the S4 of a monomer with its S5 helix, as well as at the interface of the S5 helix of one monomer and the S1 helix of the neighbouring monomer, in agreement with the SILCS results (Figs. S4, 2a). LIN interacts primarily at the S5-S1 interface. We identified several positively charged residues of the VSD (R109, R190, R192, K196) and at the S4-S5 linker of each monomer (R249 and R259) as residues engaged in hydrogen bonds with both LIN and LIN-S (Fig. S7). Q260, at the intracellular end of S5 with its side chain towards the S1 segment of the neighbouring monomer, appears to preferentially interact with LIN-S.

In summary, the MD simulations agree with previously identified PUFAs interaction sites and with the SILCS results, and identify more specific interactions for LIN-S with residues at sites 1 and 2 that are not found for the LIN molecule with a carboxylic head group alone.

### Experiments validate the importance of endocannabinoid interactions with positively charged residues in K_V_7.1

To experimentally test if LIN-S interacts with the residues in the known binding sites indicated to be important by SILCS and MD, we used the previously described mutants hK_V_7.1_K326Q/KCNE1 and hK_V_7.1_R228Q to impair interaction at site 2 and 1, respectively^46^ (note that hK_V_7.1_K326Q does not generate measurable currents without KCNE1 co-expression^46^). In line with the prediction, the K326Q mutation reduced the G_MAX_ effect caused by LIN-S (Fig. 2d), but the V_50_ effect was maintained (Fig. S8a). 10 μM of LIN-S showed impaired G_MAX_ increase in hK_V_7.1_K326Q/KCNE1 (ΔG_MAX_ = 43 ± 15%, P > 0.05, one-sample *t* test), compared to the G_MAX_ increase in hK_V_7.1/KCNE1 WT (ΔG_MAX_ = 96 ± 26%, P < 0.01, one-sample *t* test) (Fig. 2d). Moreover, in line with the prediction, the R228Q mutation reduced the shift of V_50_ caused by LIN-S (Fig. 2e) but maintained the G_MAX_ effect (Fig. S8b). 10 μM of LIN-S did not shift V_50_ of hK_V_7.1_R228Q (ΔV_50_ = −3.6 ± 3.6 mV, P > 0.05, one-sample *t* test), compared to a V_50_ shift of −33.3 ± 5.6 mV (P < 0.01, one-sample *t* test) in hK_V_7.1 WT (Fig. 2e).

We additionally explored the functional role of residues R293 and D301, which in the MD simulations form the highest number of hydrogen bonds with LIN-S. To this end, we made the hK_V_7.1_R293A and hK_V_7.1_D301A mutations. We co-expressed each mutant with KCNE1, because testing K_V_7.1_R293A alone produced negligible currents and K_V_7.1_D301A alone was previously reported to not generate currents.^55^ Similar to what we observed for the hK_V_7.1_K326Q/KCNE1 mutation, the G_MAX_ effect induced by LIN-S was reduced in the hK_V_7.1_R293A/KCNE1 mutant compared to the G_MAX_ effect in WT (Fig. 2f, P < 0.05 for 1 and 3 μM of LIN-S, but not for 10 μM because of larger variability, *t* test). For the hK_V_7.1_D301A/KCNE1 mutant, the G_MAX_ effect induced by LIN-S was completely abolished (ΔG_MAX_ for 10 μM = +8 ± 20%, P > 0.05, one-sample *t* test) (Fig. 2f). On the contrary, the V_50_ effect for both mutants was comparable to that for the WT channel (For hK_V_7.1_R293A/KCNE1: ΔV_50_ = −41.6 ± 6.8 mV; for hK_V_7.1_D301A/KCNE1: ΔV_50_ = −40.7 ± 3.8 mV) (Fig S8c). Hence, the experimental data further suggests that these residues are most important for the LIN-S interaction and effect at site 2. The stronger effects on G_MAX_ with the D301A mutant is in line with the residue engaging in more stable hydrogen-bond interactions with the bound LIN-S molecules at site 2.

All serine-based endocannabinoid-like compounds had prominent effects on V_50_ and current amplitude of hK_V_7.1/KCNE1 (Fig. 1), although with less prominent effects on G_MAX_ for some compounds (Fig. S1a). However, we will focus the remainder of our experiments on ARA-S, based on arachidonic acid, a fundamental lipid in cell membranes necessary for maintaining cell function and an abundant lipid acyl chain.^56^ Therefore, we also tested experimentally if K326 and R228 are important for the effect of ARA-S. Unlike what we observed for LIN-S, the G_MAX_ effect of 10 μM of ARA-S was not altered by the K326Q mutation (ΔG_MAX_ = 60 ± 24% for hK_V_7.1_K326Q/KCNE1 compared to 57 ± 24% for WT hK_V_7.1/KCNE1) (Fig. S8d, see also retained effect on V_50_ of hK_V_7.1_K326Q/KCNE1 in Fig. S8e), which could be due to the generally less robust G_MAX_ effect of ARA-S (Fig. S1d) or the contribution of other residues at this site (see the Discussion). On the other hand, in agreement with the data for LIN-S, the R228Q mutation reduced the shift of V_50_ caused by ARA-S. 10 μM of ARA-S did not shift V_50_ of hK_V_7.1_R228Q (ΔV_50_ = −4.9 ± 2.8 mV, P > 0.05, one-sample *t* test), compared to a V_50_ shift of −39.1 ± 3.0 mV (P < 0.0001, one-sample *t* test) in hK_V_7.1 WT (Fig. 2e, see also the less robust effect on G_MAX_ of hK_V_7.1_R228Q in Fig. S8f).

### The negative charge, but not chirality, of the endocannabinoid head group is important for effects

**Fig. 3:**
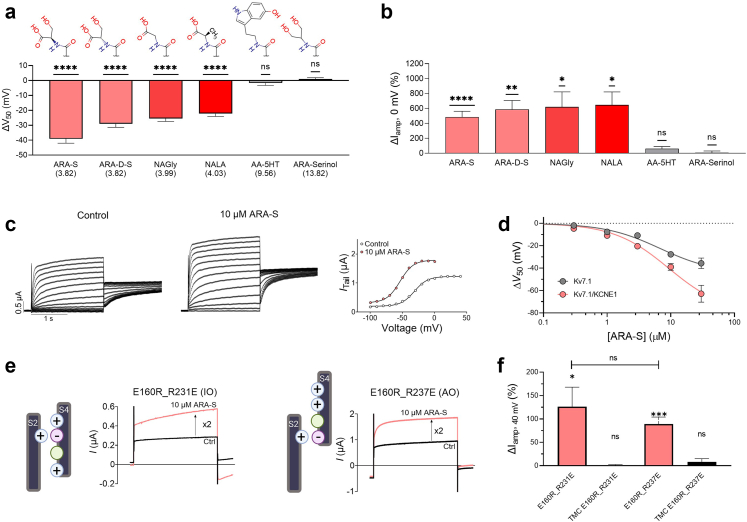
**Effect of endocannabinoid head group properties and KCNE1 for the ARA-S effect.** Effect of indicated arachidonic acid-based compounds on hK_V_7.1 or hK_V_7.1/KCNE1 expressed in *Xenopus* oocytes and studied with the two-electrode voltage clamp technique. a and b) Mean ΔV_50_ (a) and ΔI_amp_ at 0 mV (b) induced by 10 μM of ARA-S, ARA-D-S, NAGly, NALA, AA-5HT and ARA-Serinol on hK_V_7.1/KCNE1. Statistics indicate one-sample *t* test compared to a hypothetical value of 0 (i.e. no change in V_50_ or current amplitude). ∗ denotes P < 0.05, ∗∗ denotes P < 0.01, ∗∗∗∗ denotes P < 0.0001. P > 0.05 (ns). Data shown as mean ± SEM. n = 6–12. The molecular structure of the head groups of the listed compounds is shown on the top in panel A. In brackets is the calculated pKa value in solution of each compound. c) Representative traces of hK_V_7.1 currents under control conditions and in the presence of 10 μM ARA-S and corresponding G(V) curve. For this specific cell: V_50;ctrl_ = −31.3 mV, I_tailmax;ctrl_ = 1.2 μA, V_50;ARA-S_ = −53.6 mV, I_tailmax;ARA-S_ = 1.8 μA. Currents were generated in steps from −80 to +50 mV in 10 mV steps, followed by a tail voltage of −20 mV. The holding voltage was −80 mV. d) Concentration-response relation for the V_50_ effect of 10 μM ARA-S on K_V_7.1 with and without KCNE1. Best fit for K_V_7.1: EC_50_ = 6 μM, ΔV_50, max_ = −44.7 mV. Data shown as mean ± SEM; n = 4–12. e) Representative effect of 10 μM ARA-S on K_V_7.1_E160R_R231E and K_V_7.1_E160R_R237E mutants. Currents were elicited by stepping to +40 mV for 4 s followed by a tail voltage at −40 mV. Cartoon schematics illustrate IO-locked and AO-locked mutants. f) Averaged percentage of ARA-S induced current increase at the end of the pulse to +40 mV for K_V_7.1_E160R_R231E and K_V_7.1_E160R_R237E and time-match controls for respective mutants. Statistics indicate one-sample *t* test compared to a hypothetical value of 0 and student's *t* test to compare between K_V_7.1_E160R_R231E and K_V_7.1_E160R_R237E. ∗ denotes P < 0.05, ∗∗∗ denotes P < 0.001. P > 0.05 (ns). Data shown as mean ± SEM; n = 3–9.

The simulation data suggested that the negatively charged head group of endocannabinoids interact with positively charged residues on the channel. To functionally test the importance of the negative head group charge, we compared the effect of compounds that all shared an arachidonic acid tail but had different head groups. Hence, in ARA-S, we either substituted the serine head for other negatively charge head groups, such glycine (i.e. arachidonoyl glycine, NAGly) or alanine (i.e. N-arachidonoyl-L-alanine, NALA), or for uncharged head groups, such as serotonin (i.e. arachidonoyl serotonin, AA-5HT) or serinol (i.e. ARA-Serinol, an uncharged analogue of ARA-S). Please refer to Fig. 3a for head group structures and pKa values. As was observed for ARA-S, 10 μM of NAGly and NALA shifted the V_50_ of hK_V_7.1/KCNE1 (by −25.2 ± 1.9 mV and −22.2 ± 4.7 mV, respectively, P < 0.001, one-sample *t* test) and increased the current amplitude at 0 mV (by +620 ± 200% and +647 ± 174%, respectively, P < 0.05, one-sample *t* test) (Figs. 3a and b, S9a, Table 1). In contrast, 10 μM of AA-5HT and ARA-Serinol did not affect V_50_ and current amplitude at 0 mV (which remained within 1.1–1.7 mV and 7–60%, respectively) (Figs. 3a and b, S9b, Table 1). Fig. S9a summarizes the less robust G_MAX_ effects for all these compounds. The experiments substituting the ARA-S head support a critical role of the negatively charged head group of endocannabinoids to allow for prominent effects on hK_V_7.1/KCNE1 and show that the head group of ARA-S can be substituted with other negatively charged head groups with retained effects on hK_V_7.1/KCNE1.

To test if the ARA-S effect is also sensitive to the chirality of the head group, we used the unnatural D enantiomer of ARA-S (ARA-D-S, head group structure in Fig. 3a). 10 μM of ARA-D-S induced overall comparable effects to that of the natural L enantiomer of ARA-S (ΔV_50_ = −29 ± 2.3 mV; ΔI_amp_ = +587 ± 119%, Figs. 3a and b, S9a–c, Table 1). Thus, the negative charge, which is linked to the pKa value, but not chirality, of the head group impacts the ability of endocannabinoids like ARA-S to facilitate activation of hK_V_7.1/KCNE1. Moreover, the importance of the negative charge provides a mechanistic understanding why 2-AG, AEA, and NADA do not activate hK_V_7.1/KCNE1, as none of these compounds are negatively charged.

### The ARA-S effect is not altered by KCNE1

Previous work have shown that KCNE1 impairs the effect of PUFAs on K_V_7.1 by decreasing the local pH near the channel, which promotes PUFA protonation and rendering a larger fraction of PUFAs uncharged and unable to affect K_V_7.1/KCNE1.^42^^,^^47^ To more extensively assess if the KCNE1 subunit alters the ARA-S effect, we compared, side-by-side, the effect of different concentrations of ARA-S on hK_V_7.1 alone (i.e., without hKCNE1 co-expressed) to the effect on hK_V_7.1/KCNE1. As mentioned above, 10 μM of ARA-S shifted V_50_ of hK_V_7.1 by −28 ± 2 mV and increased G_MAX_ by 94 ± 25% (representative example in Fig. 3c), which was comparable to the effect previously reported on hK_V_7.1 alone^32^ and comparable to the ARA-S effect on hK_V_7.1/KCNE1 (Figs. 3d, S10a). Moreover, the ARA-S effect on hK_V_7.1 showed an overall comparable concentration response relationship to that on hK_V_7.1/KCNE1 (Figs. 3d, S10a), with the magnitude of the V_50_ effect deviating only at the highest ARA-S concentrations for which the effect on hK_V_7.1 showed sign of saturation, whereas the effect on hK_V_7.1/KCNE1 did not. A comparable effect on hKv7.1 and hKv7.1/KCNE1 was also found for LIN-S (Fig. S10b and c).

Cui and co-workers have previously shown that the propensity of hK_V_7.1 and hK_V_7.1/KCNE1 to open from different conformational states can be used to further compare the effect of compounds on hK_V_7.1 with and without hKCNE1 co-expressed.^57^ During membrane depolarization, the S4 first moves one step upward from its resting state to an intermediate state to trigger channel opening from the intermediate-open (IO) state. S4 then moves a second step to an activated state to trigger channel opening from the activated-open (AO) state. hKv7.1 alone can conduct K^+^ currents at the IO state, whereas hK_V_7.1/KCNE1 conducts K^+^ currents at the AO state.^58^ Cui and co-workers have demonstrated that the IO and AO states are isolated in the double-mutant hK_V_7.1 channels hK_V_7.1_E160R_R231E and hK_V_7.1_E160R_R237E, respectively (illustrated in Fig. 3e).^58^ To study the ability of ARA-S to increase the current of channels locked in these functional states, we tested the effect of 10 μM of ARA-S on hK_V_7.1_E160R_R231E (IO state) and hK_V_7.1_E160R_R237E (AO state) using a pulse protocol similar to previous studies.^57^ In response to a depolarizing pulse to +40 mV, ARA-S increased the steady-state current amplitude of both mutant channels (Fig. 3e). The average increase in current amplitude was 126 ± 42% and 89 ± 15%, respectively, for hK_V_7.1_E160R_R231E (IO state) and hK_V_7.1_E160R_R237E (AO state) (Fig. 3f). We note that the tail current of hK_V_7.1_E160R_R231E tended to be inward after ARA-S application. This could be because of altered relative permeability of different ions induced by ARA-S interaction at site 2, with increased Na^+^ contribution, as previously suggested for another lipid compound on hK_V_7.1 and hK_V_7.1/KCNE1.^46^ However, as we cannot completely exclude the possibility that ARA-S affects also endogenous currents, we determined the effect of ARA-S on the IO and AO mutants at +40 mV, at which, in water-injected oocytes, we did not observe ARA-S effects on endogenous currents. In summary, ARA-S facilitates activation of hK_V_7.1 with and without hKCNE1, this effect is not altered by the KCNE1 subunit, and ARA-S increases the current amplitude in both the IO and AO state.

### ARA-S cannot substitute for PIP_2_

**Fig. 4:**
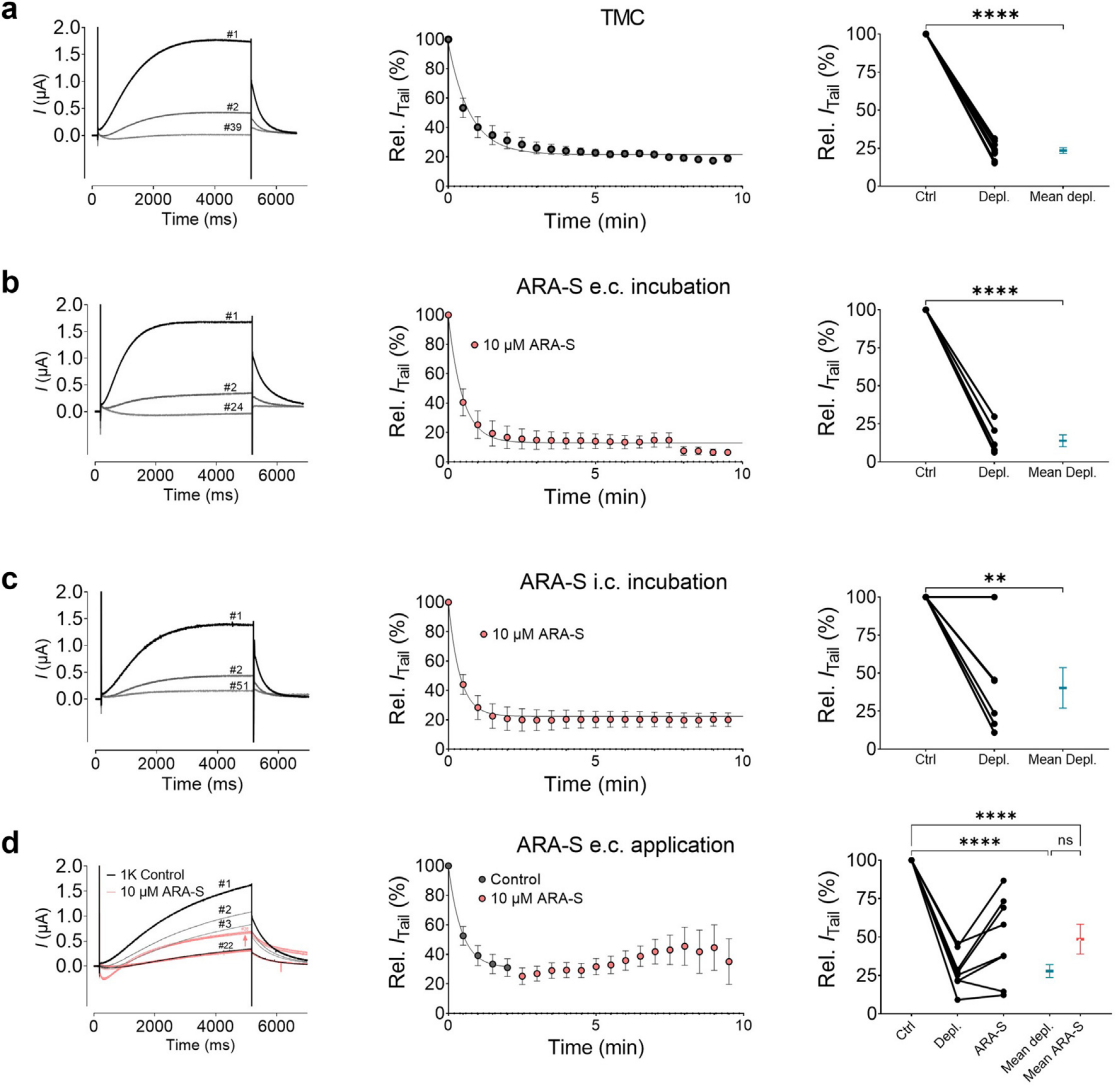
**ARA-S does not substitute for PIP**_**2**_**.** Assessment of the ability of ARA-S to compensate for PIP_2_ depletion, tested on hK_V_7.1/KCNE1 expressed in *Xenopus* oocytes and studied with the two-electrode voltage clamp technique. a) Time-match control of PIP_2_ depletion performed in oocytes co-injected with K_V_7.1/KCNE1 and Ci-VSP. PIP_2_ depletion was achieved by activating the VSP by a test pulse at +40 mV for 5 s, followed by a tail pulse at −40 mV, every 30 s. *Left*: Representative hK_V_7.1/KCNE1 current traces upon depletion. *Middle*: Averaged time-course of decay of normalized tail current. Curve show exponential fit decay; *Right*: Summary of relative tail current for each oocyte and mean value (blue). b and c) Same as in a but for oocytes incubated in (b) 10 μM extracellular or (c) intracellular ARA-S. d) Same as in a but with extracellular perfusion of 10 μM ARA-S at time points indicated by red symbols. Statistics indicate student's *t* test or one–way ANOVA. ∗ denotes P < 0.05, ∗∗ denotes P < 0.01, ∗∗∗ denotes P < 0.001, ∗∗∗∗ denotes P < 0.0001. P > 0.05 (ns). Best fit for exponential decay: panel a: tau = 0.74 min, plateau = 22%; panel b: tau = 0.48 min, plateau = 13%; panel c: tau = 0.38 min, plateau = 22%, note that the outlier (no depletion) shown in the summary of relative tail current has been excluded from the averaged time-course of decay of normalized tail current; panel d: tau = 0.44 min, plateau = 31%. Data shown as mean ± SEM; n = 6–9.

PIP_2_, a phospholipid present in the inner leaflet of the cell membrane, is necessary for hK_V_7.1/KCNE1 function by mediating electromechanical VSD to PD coupling.^37^^,^^41^ Previous work has shown that specific compounds that mimic PIP_2_ properties can act as a substitute for PIP_2_ and thereby compensate for PIP_2_ depletion.^59^ Because ARA-S shares important properties with PIP_2_, such as the lipid tail and negatively charged head group, and showed possible interactions in the inner leaflet in the simulations, we tested whether ARA-S can compensate for PIP_2_ depletion. To deplete PIP_2_ from the membrane, we followed previous protocols by co-expressing the voltage-dependent phosphatase Ci-VSP with hK_V_7.1/KCNE1 and activated the Ci-VSP with a consecutive depolarizing test pulse of +40 mV for 5 s followed by a tail pulse at −40 mV every 30 s.^60^ In line with previous studies,^61^ time-match controls (TMC, i.e., in the absence of ARA-S) showed that hK_V_7.1/KCNE1 generated clear K^+^ currents in response to the first depolarizing pulse (black line in Fig. 4a *left panel*). As the phosphatase was activated and PIP_2_ depleted from the membrane, the initial hK_V_7.1/KCNE1 K^+^ current was gradually reduced (grey lines in Fig. 4a *left panel*) with an exponential time-course of current run-down (time-course summary Fig. 4a *middle panel*), meaning that fewer channels are able to open upon PIP_2_ depletion.

To determine if ARA-S can substitute for PIP_2_ and prevent current run-down, oocytes were preincubated either in 10 μM extracellular (Fig. 4b) or intracellular (Fig. 4c) ARA-S (see SI Appendix Methods for details) prior to PIP_2_ depletion. By activating the phosphatase in these preincubated oocytes, we would expect ARA-S to alter the time-course and/or extent of PIP_2_ depletion if ARA-S was able to substitute for PIP_2_. However, the results showed that ARA-S did not alter the time-course and extent of PIP_2_ depletion, compared to the time-match control experiments (Fig. 4b and c). The only exception was one cell for intracellular preincubation, which did not show any sign of PIP_2_ depletion (Fig. 4c); the reason remains unknown. These results suggest that ARA-S is not able to substitute for PIP_2_ in mediating VSD to PD coupling. However, in cells with notable K^+^ currents subsequent to PIP_2_ depletion, ARA-S can still act on available channels to increase K^+^ currents. This is shown in experiments in which PIP_2_ was first depleted in the absence of ARA-S, followed by extracellular application of 10 μM ARA-S (Fig. 4d). In cells with close to complete PIP_2_ depletion, ARA-S was unable to clearly increase the current amplitude (Fig. 4d, *right panel*). However, in oocytes with incomplete PIP_2_ depletion, ARA-S was able to partially recover the initial current amplitude (representative example, Fig. 4d, *left panel*). Combined, this suggests that ARA-S cannot substitute for PIP_2_ by binding to the PIP_2_ site to prevent current run-down. However, ARA-S can increase the current of the fraction of channels in which PIP_2_ is still present. Therefore, to some extent, it can functionally compensate for the reduced current caused by incomplete PIP_2_ depletion.

### ARA-S facilitates activation of a phosphomimetic mutant hK_V_7.1/KCNE1 channel

**Fig. 5:**
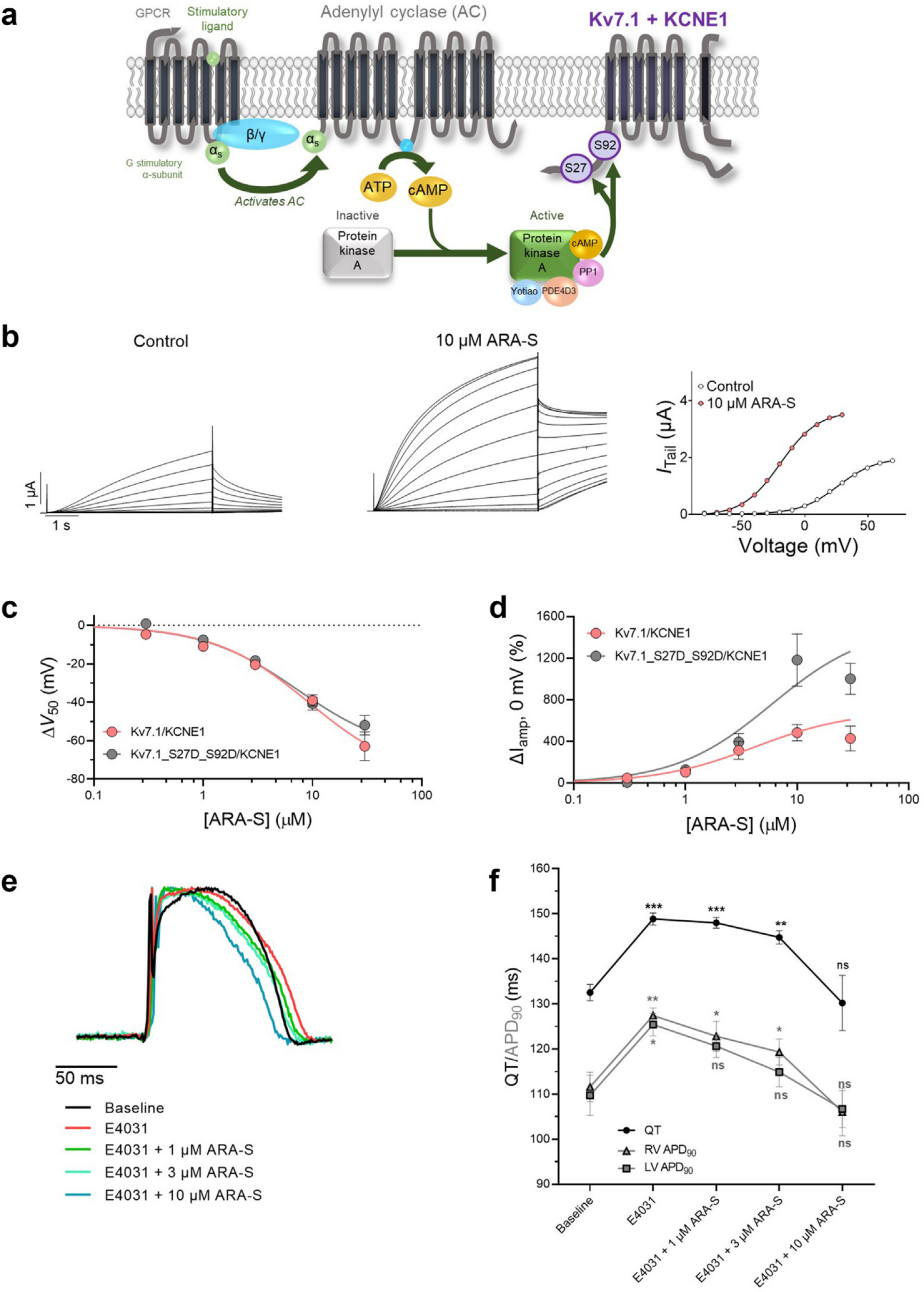
**ARA-S has preserved effect on a phosphomimetic mutant of hK**_**V**_**7.1/KCNE1 and restores action potential duration and QT interval in guinea pig hearts.** Assessment of the ability of ARA-S to act under more physiologically relevant conditions, such as under conditions mimicking the adrenergic state of hK_V_7.1/KCNE1 and in isolated guinea pig hearts, studied with electrophysiological approaches. a) Cartoon schematics of the β-adrenergic activation mechanism leading to phosphorylation of the hK_V_7.1/KCNE1 channel in two residues of the N terminus, S27 and S92. b) Representative traces of mutant hK_V_7.1_S27D_S92D/KCNE1 currents under control conditions and in the presence of 10 μM ARA-S and corresponding G(V) curve. Curves in the G(V) plot (right) represent Boltzmann fits. For this specific cell: V_50;ctrl_ = +26.3 mV, I_tailmax;ctrl_ = 1.9 μA, V_50;ARA-S_ = −19.6 mV, I_tailmax_ = 3.6 μA. c and d) Concentration-response relation for (c) ΔV_50_ and (d) ΔI_amp_ at 0 mV induced by ARA-S in hK_V_7.1/KCNE1 and hK_V_7.1_S27D_S92D/KCNE1. Best fit for ΔV_50_ on hK_V_7.1_S27D_S92D/KCNE1: EC_50_ = 7 μM, ΔV_50, max_ = −67.2 mV. Best fit for ΔI_amp_ = ambiguous. Note that the 30 μM concentration in hK_V_7.1/KCNE1 was excluded from the fit. Data shown as mean ± SEM; n = 5–13. e) Representative traces displaying the effect of E4031 on prolonging the ventricular action potential duration and the effect of ARA-S to restore the action potential duration in a concentration dependent manner in isolated guinea pig hearts. f) Summary of the changes in QT interval and action potential duration induced by E4031 alone or E4031 co-applied with indicated concentrations of ARA-S. Statistics represent two-way ANOVA with Dunnett's multiple comparisons test and indicates the difference compared to baseline. ∗ denotes P < 0.05, ∗∗ denotes P < 0.01, ∗∗∗ denotes P < 0.005, P > 0.05 (ns). Data shown as mean ± SEM; n = 6.

Physiologically, the hK_V_7.1/KCNE1 channel is most important for cardiomyocyte repolarization during adrenergic stimulation, when augmented outward K^+^ currents through hK_V_7.1/KCNE1 contributes to the shorter APD.^62^ It has been shown that adrenergic stimulation of hK_V_7.1/KCNE1 critically involves phosphorylation of two residues of the hK_V_7.1 N-terminus (S27 and S92).^63^^,^^64^ Moreover, Fedida and co-workers showed that the adrenergic state can be biophysically mimicked by the hK_V_7.1_S27D_S92D double mutant (Illustrated in Fig. 5a).^64^ We tested whether hK_V_7.1/KCNE1 activation induced by ARA-S is maintained in the hK_V_7.1_S27D_S92D/KCNE1 mutant. 10 μM of ARA-S shifted V_50_ of hK_V_7.1_S27D_S92D/KCNE1 by −40 ± 3.3 mV, increased current amplitude at 0 mV by +1182 ± 251%, and increased G_MAX_ by +118 ± 27% (Figs. 5b–d, S10d). These effects were in overall agreement with the ARA-S effect on hK_V_7.1/KCNE1 WT. Moreover, the ARA-S effect on hK_V_7.1_S27D_S92D/KCNE1 showed an overall concentration response relationship comparable to that of hK_V_7.1/KCNE1 WT (Figs. 5c and d, S10d); however, with seemingly larger effects on current amplitude and G_MAX_ at certain ARA-S concentrations (Figs. 5b, S10d). These data suggest that ARA-S augments the function of hK_V_7.1/KCNE1 also under conditions mimicking adrenergic stimulation, and that the ARA-S effect, if anything, is larger than under control conditions because the phosphomimetic mutant responded to 10 μM ARA-S with more prominent G_MAX_ increase and a larger current amplitude at 0 mV.

### ARA-S restores action potential duration and QT interval in *ex vivo* guinea pig hearts

The improved ability of hK_V_7.1/KCNE1 to conduct K^+^ currents at 0 mV in the presence of ARA-S suggests that ARA-S could have beneficial effects in conditions caused by impaired cardiomyocyte repolarization. To test this, we used an isolated guinea pig heart model of drug-induced LQTS, in which the hERG channel blocker E4031 is used to pharmacologically prolong the APD and QT interval (i.e., a pharmacological model of LQT2; see SI Appendix Methods and Liin et al.^47^ for details). Perfusing the hearts with 0.03 μM of E4031 for 20 min increased the QT interval with 16.3 ± 1.4 ms and increased the APD at 90 percent repolarization (APD_90_) of the left and right ventricle by 15.7 ± 3.7 ms and 15.8 ± 2.2 ms, respectively (Fig. 5e and f). Perfusing the hearts with 0.03 μM E4031 together with increasing concentrations of ARA-S shortened the QT interval and ventricular APD in a concentration-dependent manner. Already 1–3 μM of ARA-S (for left and right ventricle, respectively) showed significant effects in shortening the APD_90_ (P < 0.01; two-way ANOVA with Dunnett's multiple comparisons test, Fig. 5e and f). At 10 μM ARA-S, the QT interval and APD_90_ of the ventricles were normalized compared to baseline values: the QT interval was reduced by −18.7 ± 6.5 ms and APD_90_ of the left and right ventricle were reduced by −18.8 ± 3.5 ms and −21.4 ± 4.4 ms, respectively (Fig. 5e and f). These experiments show that ARA-S is able to reverse drug-induced prolongation of the QT interval and APD in isolated guinea pig hearts. In contrast, time matched control experiments using ethanol as vehicle showed no significant effect on the QT interval and APD_90_ (Fig. S11).

## Discussion

In this study, we show that specific members within the endogenous group of endocannabinoids facilitate activation of hK_V_7.1/KCNE1 expressed in *Xenopus* oocytes by shifting V_50_ towards negative voltages, increasing the current amplitude at 0 mV and increasing G_MAX_. Simulations and experiments suggest that endocannabinoids utilize previously characterized PUFA bindings sites on hK_V_7.1 and that the negative charge of the endocannabinoid head group is critical for the effect. The effect is comparable in channels with and without KCNE1 co-expression and preserved in a channel mutant mimicking the phosphorylated state of hK_V_7.1/KCNE1. Furthermore, the endocannabinoid ARA-S has beneficial effects in shortening the APD and QT interval in guinea pig hearts. Combined, these findings suggest that negatively charged endocannabinoids act noncanonically on hK_V_7.1/KCNE1 channels, raising the possibility that these compounds have protective effects in LQTS contexts.

The shift in V_50_ and increase in G_MAX_ induced by the endocannabinoids with a serine head group contribute to the overall increase of the current amplitude at voltages relevant for the cardiac action potential. These activating effects are overall similar to those induced by the structurally related family of PUFAs and PUFA analogues,^42^, ^43^, ^44^, ^45^, ^46^, ^47^ suggesting similar binding sites and mechanisms of action. PUFAs have been shown to shift V_50_ and increase G_MAX_ by binding to two distinct sites (site 1 and 2, respectively) in hK_V_7.1 to interact with the outermost S4 arginines R228 and R231 in site 1 (to facilitate outward S4 movement) and the S6 lysine K326 in site 2 (to improve K^+^ conductance).^45^, ^46^, ^47^ Of note, a comparable site 1 has been suggested to underly ARA-S effects on V_50_ of related neuronal hK_V_7 channels.^31^^,^^32^ Here, using SILCS, we found that regions of interactions surrounding such sites can be retrieved not only for LIN and for another PUFA with the carboxylic head group, i.e. ARA, but also for the corresponding endocannabinoids bearing the serine head group (LIN-S and ARA-S). However, MD simulations of K_V_7.1 in the presence of LIN and LIN-S revealed important differences between how compounds with carboxylic and serine head groups interact with K_V_7.1. Overall, higher density for LIN-S was retrieved near the previously identified sites 1 and 2, interactions which were experimentally confirmed by the impaired effect of LIN-S on G_MAX_ when mutating K326Q in site 2 and V_50_ when mutating R228Q in site 1. The analysis of hydrogen bond interactions revealed how the larger serine head group can establish hydrogen bonds with more residues compared to the carboxylic head group alone: At site 1, while LIN interacts with the previously reported R228 more than LIN-S, the larger serine head group interacts with residues deeper at the interface between the S4 segment of one monomer and the S5 segment of the neighbouring monomer (such as Y278, Y281, or K285). Similarly, at site 2, the simulations retrieve the interactions with K326 primarily for LIN, while LIN-S can interact with a larger number of residues closer to the pore domain and in the surrounding extracellular loops, particularly with D301 and R293. Thus, while sites 1 and 2 remain the primary regions of interactions of K_V_7.1 with PUFAs and endocannabinoids, distinct residues can control the effect of different compounds on modulating channel activity, which could be a possible explanation for why the hK_V_7.1_K326Q/KCNE1 mutation did not alter the G_MAX_ effect of ARA-S. The improved ability of serine compounds to interact with K_V_7.1 in simulations compared to fatty acids, despite the same negative charge in the headgroup, suggests that the larger experimental effects induced by serine compounds are not only determined by different pKa values of the head groups (about 1 pH unit lower for a serine head group compared to a carboxylic head group). It is important to note that our simulations were done on K_V_7.1 alone, without the KCNE1 subunit. This is because the structure of the K_V_7.1/KCNE1 complex has not yet been determined. The presence of KCNE1 in the simulation systems may affect endocannabinoid distribution at the K_V_7.1 channel. However, because ARA-S and LIN-S show a generally similar experimental effect on K_V_7.1 and K_V_7.1/KCNE1, we anticipate overall similar endocannabinoid interactions with and without KCNE1. The preserved ARA-S and LIN-S effect upon KCNE1 co-expression is likely due to the low apparent pKa value of these compounds, which makes them more resistant to altered local pH. In this way, endocannabinoids with low enough apparent pKa can evade the indirect KCNE1-induced protonation that has been previously shown to impair the effect of PUFAs with carboxylic head groups upon KCNE1 co-expression.^42^^,^^47^

In addition, K249 and R259 are among the residues in the simulations that in the lower leaflet establish hydrogen bonds with LIN and LIN-S. This region was also described for LIN in a previous MD simulation study but shown in experiments to not be functional for PUFAs.^45^ The natural ligand for the region near K249 is PIP_2_^37^ raising the possibility that ARA-S may contribute to electromechanical coupling from this site. However, our experiments suggest that ARA-S neither affects the time-course or extent of PIP_2_ depletion. This indicates that endocannabinoids either do not target this site or have no functional effect at the site. This is different from the hK_V_7.1/KCNE1 channel modulator CP1, which can substitute for PIP_2_ to mediate electromechanical VSD-PD coupling.^59^ Of note, we found ARA-S to functionally compensate for PIP_2_ depletion upon incomplete depletion (i.e., when there was a notable K^+^ current remaining), suggesting that ARA-S can augment hK_V_7.1/KCNE1 channel function at intermediate PIP_2_ levels when there are some functional channels available for ARA-S to act on. In our hands, AEA and 2-AG did not have effects in the hK_V_7.1/KCNE1 channel, likely because they do not have a negatively charged head group. This is in contrast to TRPV1, which AEA and 2-AG directly activate by binding to a tunnel formed by the S1-S4 region and the vanilloid-binding pocket located between S3-S4 of one monomer and S5-S6 of an adjacent monomer.^65^ The mechanistic differences in endocannabinoid interactions with hK_V_7.1/KCNE1 and TRPV1 highlight that endocannabinoids can have differential sites and effects on ion channels.

Endocannabinoids like 2-AG and AEA affect several cardiac ion channels, such as the potassium channels K_V_4.3 and K_V_1.5,^24^^,^^25^ and Na_V_ and L-type Ca_V_ channels in ventricular myocytes.^26^ In this study, we provide further insights into noncanonical cardiac targets of endocannabinoids by showing that ARA-S, LIN-S, DOC-S, and NAGABA augment the function of hK_V_7.1/KCNE1. The ARA-S effects we observe on hK_V_7.1/KCNE1 in this study, with significant effects at 300 nM, occurs at similar concentrations as those previously reported for endocannabinoids on TRPV1, K_V_1.5, ASIC3, and K_V_4.3.^24^^,^^25^^,^^66^^,^^67^ Moreover, this is in the physiological concentration range reported for 2-AG in circulation, which ranges from 1 to 400 nM in healthy individuals and can increase further during stress and pathology.^50^ Although the less-characterized endocannabinoids, like ARA-S, are anticipated to share overall similar biosynthetic and degradatory pathways with AEA,^11^^,^^29^ there is limited knowledge about the abundance of ARA-S and many of the other endocannabinoids in different tissues, which is difficult to determine for compounds that are locally released and accumulated in membranes. Therefore, a limitation of this study is that knowledge about local concentrations at cardiomyocytes are needed to allow for an evaluation of whether ARA-S and other negatively charged endocannabinoids might have physiological or pathophysiological functions in the human heart. Moreover, although we find significant effects by sub-micromolar concentrations of specific endocannabinoids in our experiments, micromolar concentrations are used in most experiments to induce clear and robust effects, which therefore do not reflect physiological *in vivo* levels. Also, our experimental setting does not capture the complex *in vivo* regulation of endocannabinoids, which for instance involves the endocannabinoid carrier albumin.

Recent studies have demonstrated beneficial effects in a LQTS context of modulators augmenting the activity of hK_V_7.1/KCNE1. There are several compounds that restore a physiological APD and QT interval in pharmacological and/or genetic experimental models of LQTS. Most of these compounds have different mechanisms of action and binding sites compared to ARA-S. For example, the mentioned CP1 compound, in contrast with ARA-S, substitutes for PIP_2_ after PIP_2_ depletion and has been suggested to bind to the PIP_2_ pocket to mediate VSD-PD coupling.^59^ The small-molecule compound C28 enhances current amplitude in the channel only with the VSD arrested in the activated state,^68^ and the small-molecule compound ML277 augments the hK_V_7.1/KCNE1 complex, in a stoichiometry dependent manner, by specifically enhancing the current of the AO state when binding to its pocket located on the intracellular side.^57^^,^^69^ Furthermore, hK_V_7.1 antibodies increase channel open time and open probability by targeting an extracellular region close to the selectivity filter of hK_V_7.1/KCNE1.^70^ Thus, endocannabinoids, PUFAs, PUFA analogues, and the above listed hK_V_7.1/KCNE1 channel modulators make a set of chemically varied modulators with APD and QT shortening effects, acting through diverse mechanisms. This could be utilized in the development of future targeted treatment of LQTS, in which the preferred mode of modulation would be guided based on the underlying cause of the disease.

To conclude, this study shows that specific members within the endocannabinoid family target the cardiac hK_V_7.1/KCNE1 channel. The pronounced activation of hK_V_7.1/KCNE1 and the shortening of the APD and QT interval in guinea pig hearts by ARA-S highlight ARA-S and other negatively charged endocannabinoids as putative endogenous modulators of hK_V_7.1/KCNE1 and as model compounds for drug development.

## Contributors

All authors read and approved the final version of the manuscript. I.H-I, M.A.S, S.L, J.N, S.I.L performed electrophysiology experiments and analysed related data. L.M.C-G, V.C, S.Y performed simulations and analysed related data. I.H-I, V.C, M.A.S, S.Y, S.Y.N, B.H.B, D.P.T, S.I.L contributed to study design. All authors contributed to manuscript writing. I.H-I and S.I.L verified the underlying data.

## Data sharing statement

All numerical data of the work are provided in the main figures, supplementary figures, and tables. Any additional information, including trajectories, are openly available upon request to the corresponding author (e-mail: sara.liin@liu.se).

## Declaration of interests

A patent application (#62/032,739) including a description of the interaction of charged lipophilic compounds with the K_V_7.1 channel has been submitted by the University of Miami with S.I.L. identified as one of the inventors. The other authors have no conflict of interest to declare.

## Figures and Tables

**Hiniesto-Inigo et al R2 Figure S1:**
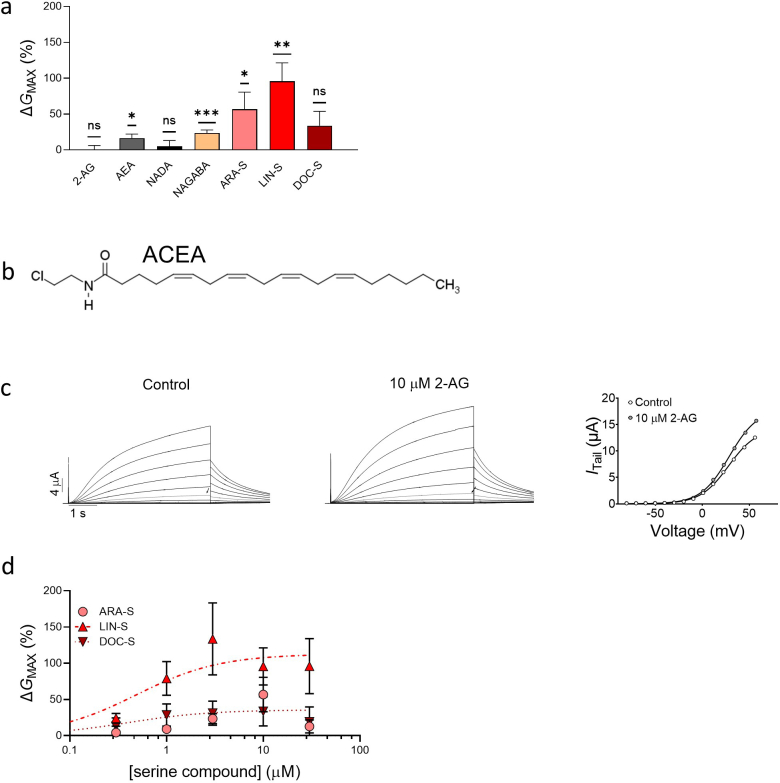


**Hiniesto-Inigo et al R2 Figure S2:**
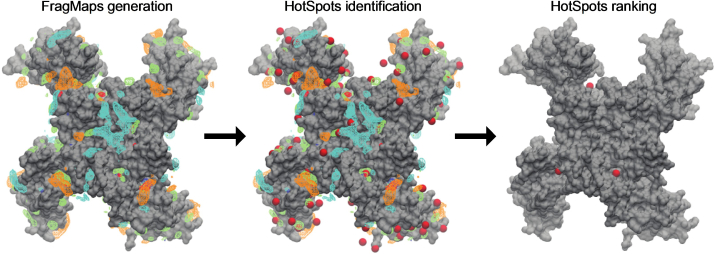


**Hiniesto-Inigo et al R2 Figure S3:**
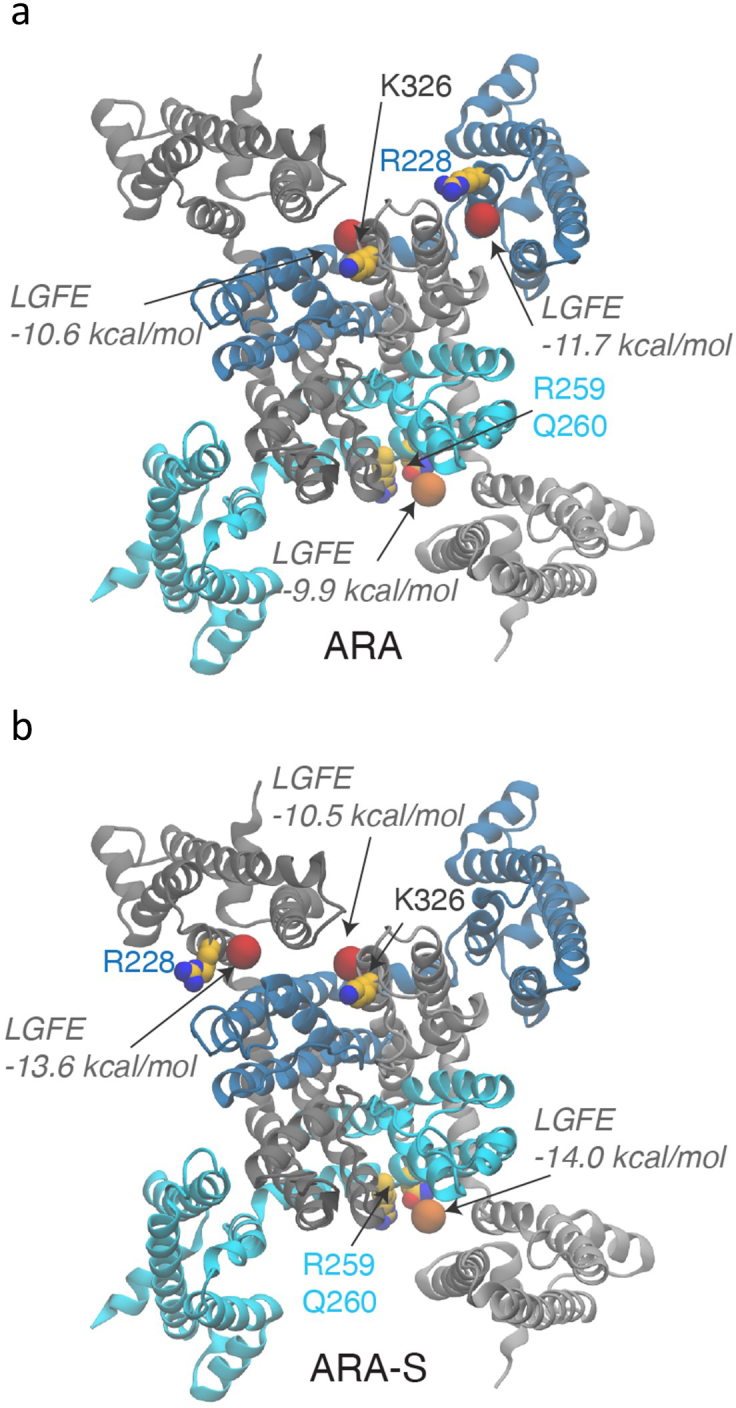


**Hiniesto-Inigo et al R2 Figure S4:**
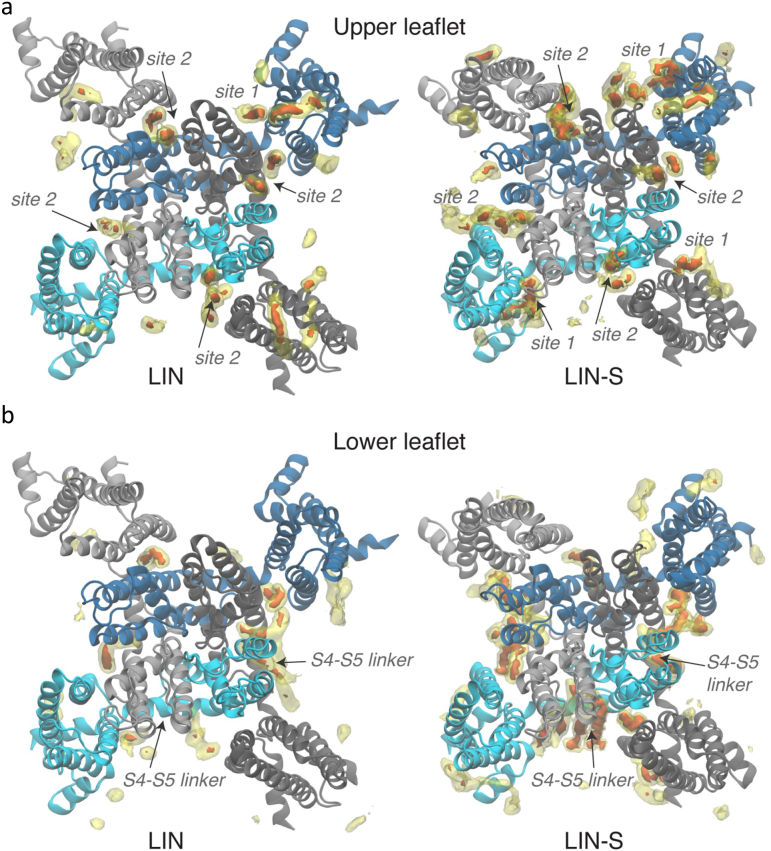


**Hiniesto-Inigo et al R2 Figure S5:**
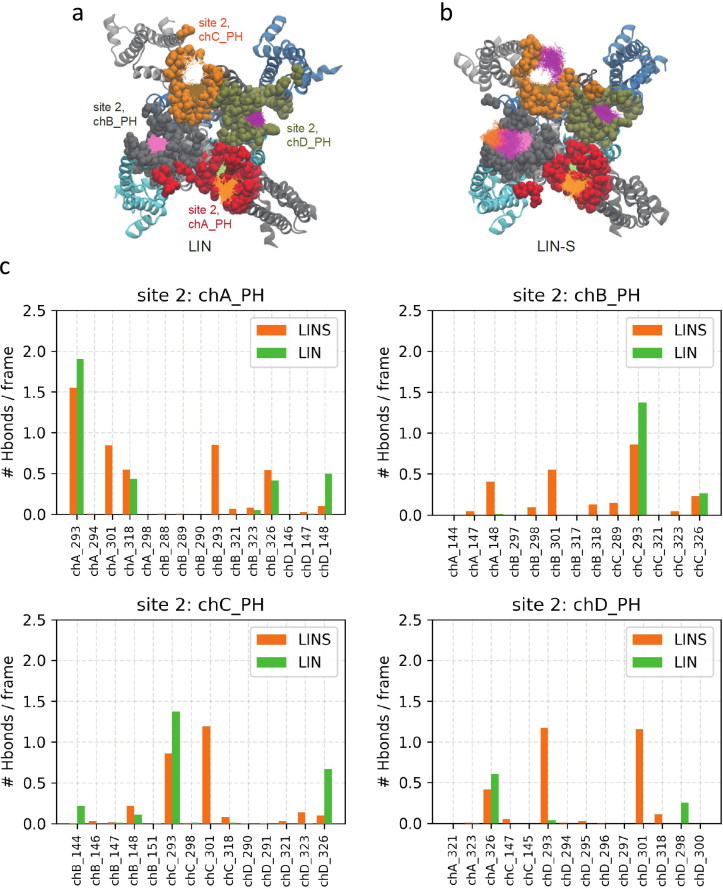


**Hiniesto-Inigo et al R2 Figure S6:**
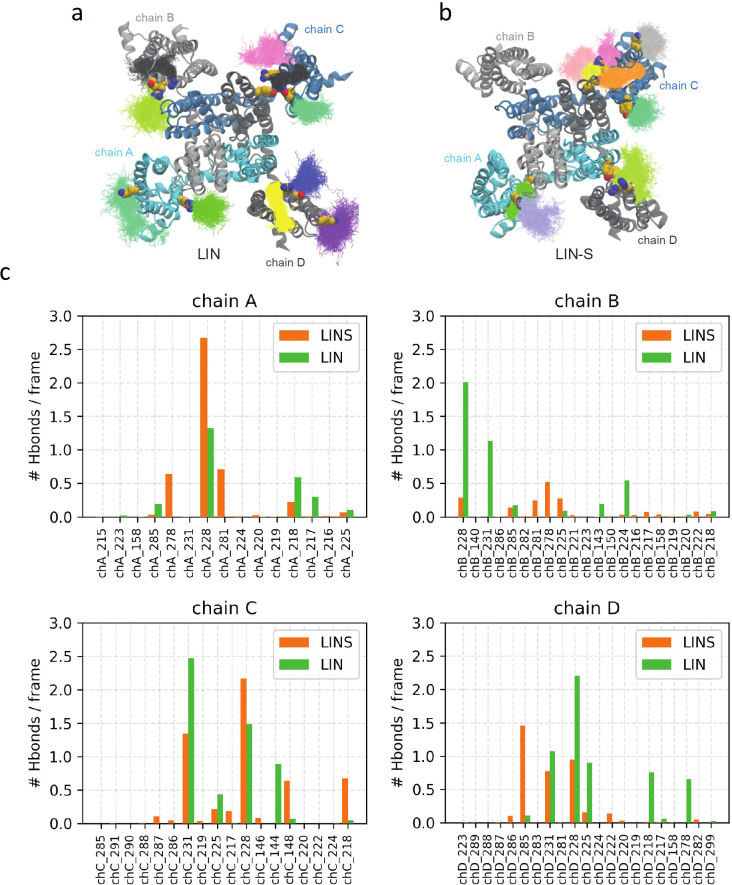


**Hiniesto-Inigo et al R2 Figure S7:**
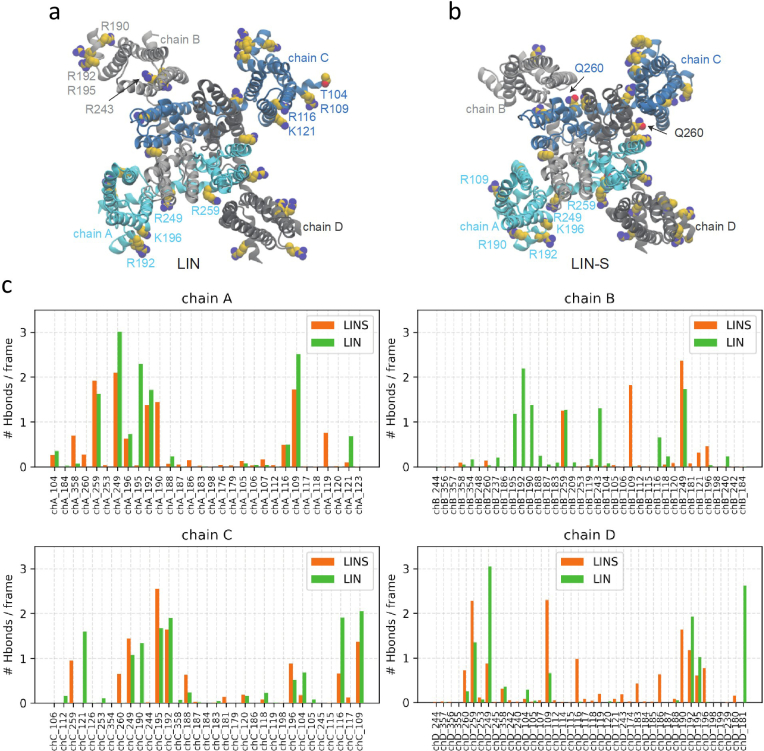


**Hiniesto-Inigo et al R2 Figure S8:**
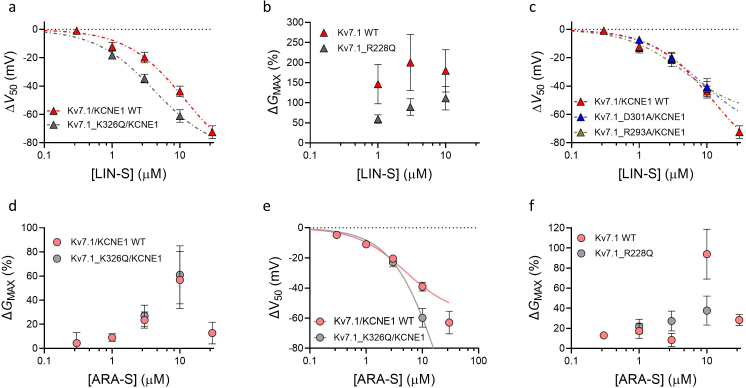


**Hiniesto-Inigo et al R2 Figure S9:**
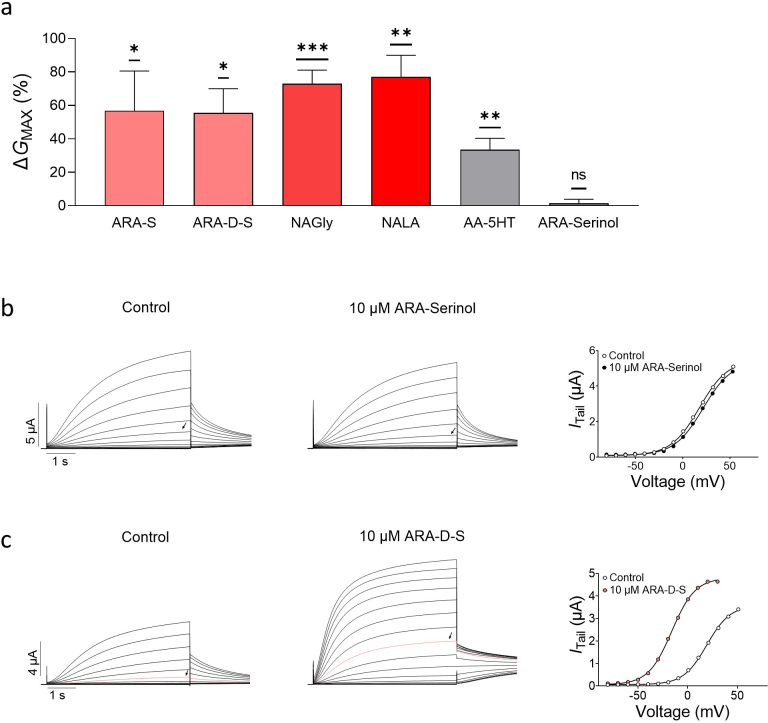


**Hiniesto-Inigo et al R2 Figure S10:**
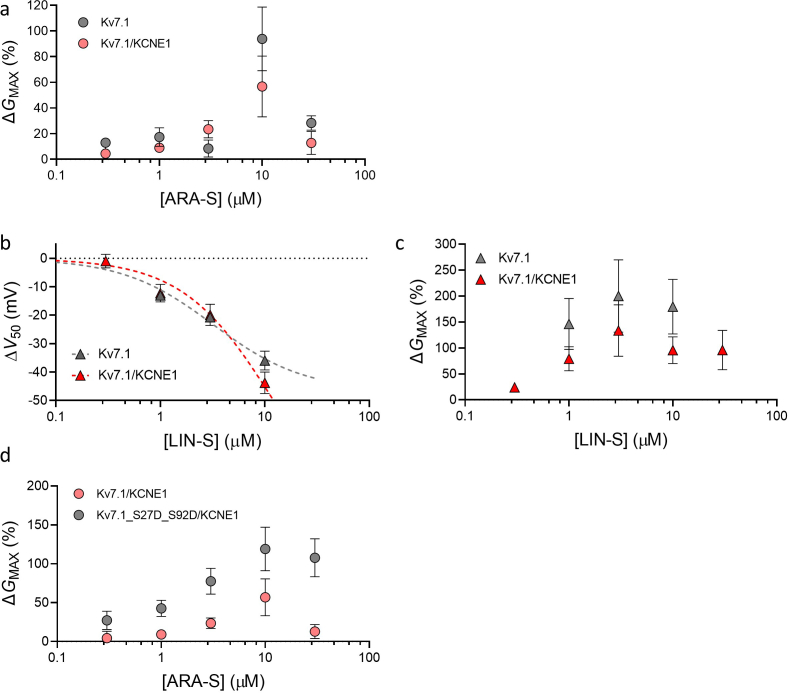


**Hiniesto-Inigo et al R2 Figure S11:**
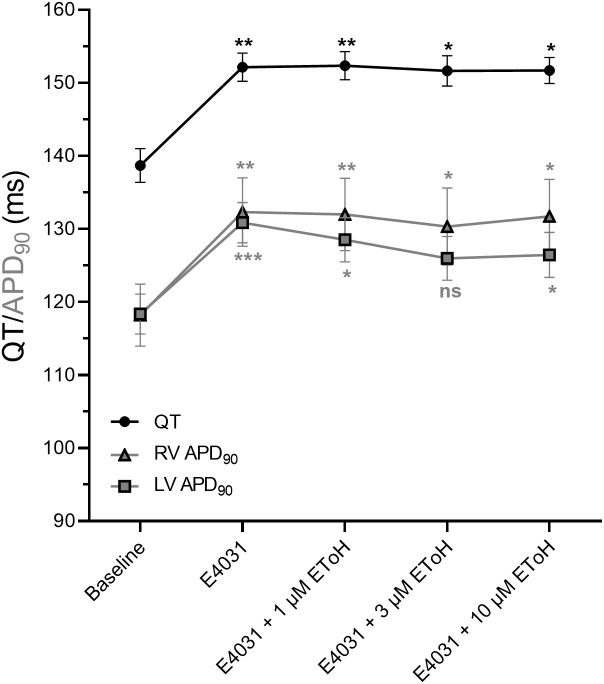


## Appendix A Supplementary data

Hiniesto-Inigo et al R2 SI Clean
