# Supplementary material for: Endocannabinoids enhance hKV7.1/KCNE1 channel function and shorten the cardiac action potential and QT interval
Source: eBioMedicine. 2023 Feb 14;89:104459. doi: 10.1016/j.ebiom.2023.104459 (PMC9958262; doi:10.1016/j.ebiom.2023.104459)
Supplement: Hiniesto-Inigo et al R2 SI Clean [file mmc1.pdf]

Supporting Information

Endocannabinoids enhance hK_V7.1/KCNE1 channel function and shorten the cardiac action potential and QT interval

Irene Hiniesto-Iñigo, Laura M. Castro-Gonzalez, Valentina Corradi, Mark A. Skarsfeldt, Samira Yazdi, Siri Lundholm, Johan Nikesjö, Sergei Yu Noskov, Bo Hjorth Bentzen, D. Peter Tieleman, Sara I. Liin

Supporting Material and Methods

Ethics

Xenopus laevis experiments were approved by the Linköping Animal Care and Use Committee (Permit #1941) and conform to national and international guidelines. Guinea pig experiments were performed at the Department of Biomedical Sciences, University of Copenhagen, Denmark and done according to the Danish guidelines for animal experiments under license 2017-15-0201-01296.

Test compounds

All chemicals were purchased from Sigma-Aldrich (Stockholm, Sweden) if not stated otherwise. 2-arachidonoyl glycerol (2-AG, Cayman #62160), arachidonoyl ethanolamide (AEA, also called anandamide, Cayman #90050), N-arachidonoyl-L-serine (ARA-S, Cayman #10005455), N-arachidonoyl dopamine (NADA, Cayman #90057), N-arachidonoyl-γ-aminobutyric (NAGABA, Cayman #90067), arachidonoyl serinol (ARA-Serinol, Cayman #62170), arachidonoyl alanine (NALA, Cayman #90065), arachidonoyl glycine (NAGly, Cayman #90051) and arachidonoyl serotonin (AA-5HT, Cayman #70665) were bought from Cayman Chemicals (MI, USA). Arachidonyl-2´-chloroethylamide (ACEA, Tocris #1319) was bought from Tocris Bioscience (Bristol, UK). N-docosahexaenoyl-L-serine (DOC-S), N-linoleic-L-serine (LIN-S), and N-arachidonoyl-D-serine (ARA-D-S) were synthesized *in house* as described previously.[1] Stock solutions of 25-100 mM were prepared in 99.5% EtOH and stored at -20°C except for 2-AG, NAGly and AA-5HT which were stored at -80°C. The final test solution was prepared on the day of the experiment by diluting the stock compound in control solution to reach the required concentration.

Constructs and mutagenesis

Human $K_V7.1$ (GenBank accession no. NM_000218) and KCNE1 (GenBank accession no. NM_000219) were in expression plasmids pGEM. Mutations were introduced through site-directed mutagenesis (QuikChange II XL, with 10 XL Gold cells; Agilent Technologies, Kista, Sweden) and confirmed by

sequencing at the Linköping University Core Facility. cRNA was linearized using T7 mMessage mMachine transcription kit (Ambion/Invitrogen). Ci-VSP (GenBank accession no. NC_020167.2) was in expression plasmid pSD64TF. cRNA was linerarized using SP6 mMessage mMachine transcription kit (Ambion/Invitrogen). The RNA concentration was quantified using spectrophotometry (NanoDrop 2000c; Thermo Fisher Scientific).

***Xenopus laevis* oocyte experiments**

Xenopus oocytes were surgically isolated at Linköping University or purchased from EcoCyte Bioscience (Dortmund, Germany). A total of 28 adult (older than 12 month) female *Xenopus laevis* frogs had been previously obtained from Nasco (WI, USA). The animals were housed together in groups of five in purposed tanks, with nests and environmental enrichment. They were kept in a water temperature of 17-19°C, with a pH of 6.4-7.8 and a conductivity of 400-1000 µS. The frogs were monitored daily by trained caretaker and fed twice a week. During surgery, frogs were anesthetized with 1.4 g/l Ethyl 3-aminobenzoate methanesulfonate salt (Tricaine) and the depth of anesthesia was monitored by checking for reflexes when the paws were pinched. Local analgesics (5mg/mL Marcain; Astra Zeneca, Sweden and 2% Xylocain gel, Aspen Nordic, Denmark) were used post-surgically to relief pain. No animals were excluded before surgery. Following the animal experimental license, the human endpoints were euthanasia with Tricaine in association with the sixth surgery, or if any animals were not thriving or were sick. Isolated *Xenopus* oocytes were injected with 50 nl of $hK_V7.1$ RNA (25 ng RNA) for expression of $hK_V7.1$ alone or 12.5 ng $hK_V7.1$ RNA and 7.5 ng KCNE1 RNA for co-expression of $hK_V7.1$/KCNE1. For PIP_2 depletion experiments, 12.5 ng $hK_V7.1$ + 7.5 ng KCNE1 RNA were co-expressed with 1.5-4.5 ng RNA of Ci-VSP. The oocytes were incubated at 8°C or 16°C for 2-5 days before performing two-electrode voltage clamp experiments in Modified Barth's Solution consisting of (in mM): 88 NaCl, 1 KCl, 2.4 $NaHCO_3$, 0.33 $Ca(NO_3)_2$, 0.41 $CaCl_2$, 0.82 $MgSO_4$, 15 HEPES and 2.5 pyruvate, with pH set to 7.6 using NaOH. The two-electrode voltage clamp recordings were performed at room temperature using a Dagan CA-1B amplifier (Dagan, MN, USA) or an

AxoClamp 900A amplifier (Molecular Devices, CA, USA). Pulled microelectrodes (0.4-1.5 MΩ, World Precision Instruments Inc, FL, USA) were filled with 3 mM KCl. Whole-cell K^+ currents were sampled using Clampex (Molecular Devices Ltd, Wokingham, UK) at 5 kHz and filtered at 500 Hz. The holding voltage was generally set to -80 mV. Activation curves were generally generated in steps between -80 and +70 mV in increments of 10 mV (2-s duration for $hK_V7.1$ or 5-s duration for $hK_V7.1$/KCNE1). The tail voltage was set to -20 mV. For mutants locked at open states, the holding voltage was set to -80 mV. Activation curves were generated in steps between -120 and +40 mV in increments of 10 mV (4-s duration). The tail voltage was set to -40 mV. The control solution contained (in mM): 88 NaCl, 1 KCl, 15 HEPES, 0.4 $CaCl_2$, and 0.8 $MgCl_2$, with pH set to 7.4 using NaOH. Control solution or control solution supplemented with test compounds was continuously perfused through the recording chamber (1 mL/min) using a pump (Harvard Apparatus MP II, CMA Microdialysis, Kista, Sweden or Minipuls 3 peristaltic pump, Gilson, WI, USA). Each test substance was applied until a stable effect on current amplitude was observed or for a minimum of 5 min, monitored by running an application protocol stepping from a holding voltage of -80 mV to a test voltage of 0 mV every 10 seconds. The tubing system and recording chamber were cleaned between cells with 70% EtOH and distilled H_2O.

For PIP_2 depletion experiments, PIP_2 was depleted by activating Ci-VSP by stepping to depolarizing pulses to +40 mV for 5 seconds from a holding voltage of -80 mV every 30 second. Subsequent tail currents were recorded at -40 mV. In one set of PIP_2 depletion experiments, ARA-S was applied through extracellular incubation prior to experiments, in which oocytes were incubated for 15 min – 1 h in 10 µM of ARA-S prior to the PIP_2 depletion experiments (10 µM of ARA-S was also present extracellularly throughout the experiment). These experiments are referred to as "ARA-S extracellular incubation". In another set of PIP_2 depletion experiments, ARA-S was applied through intracellular incubation prior to experiments, in which oocytes were injected with 50 nl of 200 µM ARA-S 2-10 minutes prior to experiments, giving an approximate intracellular concentration of 10 µM

of ARA-S (assuming 1 µl of oocyte volume,[2]). These experiments are referred to as "ARA-S intracellular incubation".

Electrophysiological analysis

Electrophysiological analysis was performed in GraphPad Prism 9 (GraphPad Software Inc., CA, USA). To quantify the voltage dependence of channel opening, tail currents were measured shortly after stepping to the tail voltage and plotted against the preceding activation voltage. A Boltzmann function was fitted to the data to generate the conductance versus voltage (G(V)) curve:

$$G(V) = G_{MIN} + (G_{MAX} - G_{MIN}) \Big/ \left\{1 + \exp\left[\frac{V_{50} - V}{s}\right]\right\}, \qquad (1)$$

where G_{MIN} is the minimum conductance, G_{MAX} the maximum conductance, V_{50} the midpoint (i.e., the voltage at which the conductance is half the maximal conductance determined from the fit), and s the slope of the curve. The difference in V_{50} induced by the compound in each oocyte (i.e., ΔV_{50}) was calculated to quantify the shift in the voltage dependence for channel opening. The difference in G_{MAX} induced by the compound in each oocyte (i.e., ΔG_{max}) was calculated to quantify the change in the maximum conductance. The difference in steady state current amplitude induced by the compound in each oocyte (i.e., ΔI_{amp}) was calculated at the end of the activation pulse to 0 mV, a physiological relevant voltage,[3,4] and normalized to the current amplitude in control solution. To estimate the effect of ARA-S in the intermediate-open or activated-open states, ARA-S was perfused once the current in the control solution was stable using a protocol that stepped the voltage to +40 mV for 4 seconds. The difference in current amplitude induced by ARA-S in each oocyte was calculated at the end of the pulse. Only cells with a stable leak of less than 500 nA were selected. The results are shown in percentage (0% means no change).

To plot the concentration dependence of the compound-induced effect as a function of the compound concentration, the following concentration-response curve was fitted to the data:

$$\Delta\text{Effect} = \Delta\text{effect}_{MAX} / \left[1 + \left(\frac{EC_{50}}{C}\right)^{H}\right], \tag{2}$$

where Δeffect$_{MAX}$ is the maximal shift in V_{50}, change in current amplitude or change in G_{MAX}, EC_{50} the concentration needed to cause 50% of the maximal effect, and H the Hill coefficient (set to 1).

To quantify PIP_2 depletion, the following exponential time course of run-down was used:

$$\text{Rel } I_{Tail} (\%) = (100 - \text{Plateau})*\exp(-K*\text{time}) + \text{Plateau} \tag{3}$$

All the oocytes in time-match control experiments showed PIP_2 depletion with an average of 23% of the initial tail current remaining after depletion (Fig. 4A *right panel*), which is less depletion compared to some studies but comparable to other studies.[5-7]

SILCS

SILCS setup. We used the Site-Identification by Ligand Competitive Saturation (SILCS) software[8] to identify putative binding sites for LIN, LIN-S, ARA and ARA-S in K_V7.1. SILCS combines the Grand Canonical ensemble-based Monte Carlo (GCMC) sampling with nanosecond-length atomistic MD simulations, to account for protein flexibility in the search the possible regions of interactions, and improve configurational sampling of probe species. Explicit water molecules (55 M) and a collection of probe molecules (0.25M) representing apolar (aromatic and aliphatic) groups, hydrogen bond donors and acceptors, and charged functional groups compete during the simulations for binding sites on the protein to generate free energy maps. The details of the SILCS procedure have been described elsewhere.[8-11] As a starting structure, we used the *Xenopus laevis* K_V7.1 structure, solved

by cryo-EM at 3.7 Å (PDB: 5VMS).[12] In describing the results, the human $K_V7.1$ numbering is used. The protein was embedded in a POPC: cholesterol (10:1 ratio) membrane, with six PIP_2 lipids in the lower leaflet, using CHARMM-GUI.[13] SILCS protocol includes the generation of ten different systems, where the protein-lipid complex are solvated with explicit water molecules and eight types of probe molecules (i.e. propane, benzene formamide, imidazole, methanol, acetaldehyde, methyl-ammonium and acetate), randomly distributed for each of the ten systems. SILCS represents lipids and protein using the CHARMM36[14] and CHARMM36m[15] forcefield, respectively, and water molecules with the CHARMM TIP3P model.[16] Simulations parameters for the probe molecules were obtained with the CHARMM General Force Field (CgenFF).[17,18] The SILCS simulations were performed using a time step of 2 fs, and temperature and pressure were kept at 298 K and 1 bar using the Nose-Hoover thermostat[19,20] and the Parrinello-Rahman barostat,[21] respectively. For the minimization and equilibration steps we used the default parameters, described in Ustach et al.[11] During the production runs, harmonic restraints were applied only to the α-carbon atoms of the protein, with a force constant of 0.12 kcal/mol* Å.^{2} We performed 100 cycles of GCMC/MD comprising 200,000 GCMC steps and 1 ns of MD simulation, generating 100 ns data for each of the 10 simulation systems, for a total of 1 µs.

FragMaps. We used the previously described trajectories as input for SILCS to generate "FragMaps", energy-based 3D-maps representing the occupancy or affinity patterns of the selected probes for the protein. To obtain the FragMaps, the 3D-occupancy maps are normalized considering the distribution of each probe without the protein and a Grid Free Energy (GFE) parameter is calculated for each probe molecule using the following Boltzmann transformation on the normalized distribution:

$$GFE_{xyz}^{T} = min\left\{-RTlog_e \frac{occupancy_{x,y,z}^{F}}{< bulk\ occupancy >}\right\}$$

FragMaps were constructed for different atoms: benzene carbons, propane carbons, methanol oxygens, methanol polar hydrogens, formamide polar hydrogens, formamide oxygen, methylammonium polar hydrogens, acetaldehyde oxygens, and acetate oxygens. In addition, generic

FragMaps were generated to include apolar atoms (benzene and propane carbons), generic hydrogen bond donor atoms (formamide and imidazole polar hydrogens), and generic hydrogen bond acceptor atoms (formamide and acetaldehyde oxygens and imidazole acceptors nitrogen). Positively and negatively charged FragMaps were obtained with methylammonium nitrogen and the acetate carbonyl carbon atoms.[22] Exclusion Maps were created considering the regions of the grids where no sampling was made by the water or the solutes during the SILCS simulations.

Ligand Grid Free Energy Calculations. We used the SILCS Monte Carlo (SILCS-MC) algorithm on the pre-generated FragMaps grid for ligand scoring. In this approach, each atom in the ligands of choice is classified and associated with a FragMap type. A GFE score is assigned to each atom type based on the value of the FragMap at that position, and combined with a scale factor as previously described.[10,11] A ligand GFE (LGFE) score is then calculated based on the summation of the atomic GFE scores:

$$LGFE = \sum_{FragMaps,\ T\ atoms,\ i_T} \sum GFE^{T}_{x_i, y_i, z_i(i_T)}$$

^{i}T corresponds to the FragMaps types and GFE^{T} is the GFE for various FragMaps types. The Exclusion Map is also included with a GFE value of 1000 kcal/mol, to avoid sampling the region occupied by the protein. During SILCS-MC, ligands are sampled based on rotational, translational, and dihedral degrees of freedom. The CgenFF parametrization is employed for intramolecular energies including dihedral, vdW, and electrostatic terms in addition to the LGFE score.[10,11]

SILCS Hotspots. We used the SILCS-Hotspots module for fragment screening, to generate fragment poses with their associated LGFE scores.[9] Here, for $K_V7.1$, we used a library of four molecules: LIN, LIN-S, ARA, ARA-S. The methodology divides the full 3D space of the protein into a collection of subspaces in which each ligand is randomly positioned and docked a minimum of 1000 times to identify favoured local poses, and two clustering steps are made to identify binding sites.[9] Finally, for each ligand, a list of putative binding sites and estimated ligand-binding free energy for each site is

generated. In the case of $K_V7.1$, we partitioned the simulation box into subspaces of size 14.14 Å × 14.14 Å × 14.14 Å, and used the default parameters described in MacKerell et al.[9]

MD simulation setup

Channel preparation. The cryo-EM structure of human $K_V7.1$ solved at 3.1 Å[23] was used to build the complete transmembrane domain (residues 104–358) of the human $K_V7.1$ channel. CHARMM-GUI Martini Bilayer Maker[13] was used to prepare the system containing the protein embedded in a pure phosphatidylcholine bilayer solvated in 0.1 M KCl aqueous solution using CHARMM36 force field and TIP3P water model.[16,24,25] The system was initially subjected to steepest descent energy minimization and subsequently equilibrated for ~2 ns before starting 500 ns production simulation using Gromacs with 2-fs time steps.[26] The LINCS algorithm [27] was applied for constraining bond lengths. Electrostatic interactions were calculated with the Particle-Mesh Ewald algorithm at every step.[28] A 1.2-nm cutoff was used both for electrostatics and van der Waals interactions, with neighbours list updated every 20 steps. The simulations were performed at constant pressure of 1.0 bar with Parrinello–Rahman pressure coupling [21] and the semi-isotropic pressure scaling, time constant of 5.0 ps, and a system compressibility of $4.5e^{-5}$ bar^{-1}. The temperature of the system was maintained at 300K using the extended Nosé–Hoover thermostat.[20] The 500 ns fully equilibrated structure was used to seed the all-atom simulations in multicomponent lipid membranes containing either the PUFA LIN or endocannabinoid LIN-S.

LIN/LIN-S simulations. The relaxed structure of the human $K_V7.1$ channel was embedded into a lipid bilayer consisting of phosphatidylethanolamine:phosphatidylglycerol:cholesterol:LIN/LIN-S (POPE:POPG:CHOL:LIN/LIN-S) in a 3:1:1:1 ratio. As $K_V7.1$ requires the presence of phosphatidylinositol 4,5-bisphosphate (PIP_2) lipids for its function, PIP_2 lipids were included in the inner leaflet in every bilayer simulation. The lipid composition for these systems was as follows: 313 POPE, 104 POPG, 89 CHOL, 4 PIP_2, and 120 LIN/LIN-S. Each system was equilibrated for ~2 ns with

Gromacs[26] according to the protocol described above before production runs were performed for 5 μs.

MD simulations analyses

Density analysis. For each system, we extracted the last 1 μs of simulation time and processed the trajectory files by means of progressive fitting on the α-carbon atoms of the protein. For this analysis frames were saved every 1 ns. We then used the MDAnalysis 2.0.[29,30] LeafletFinder module to identify the ligands in the upper leaflet and in the lower leaflet, and the Density module to obtain the number density of each ligand, using a grid spacing of 1 Å. The grid center and dimensions were set to be the same for all systems. The visualization of the density was done with VMD 1.9.3,[31] using isosurfaces drawn at 0.19 and 0.09, which correspond to 40% and 20%, respectively, of the maximum density detected for the upper leaflet.

Identification of residues interacting with LIN-S or LIN at sites 2. This analysis was performed on the last 1 μs of simulation time for each system, with frames saved every 1 ns. For each system, for the ligands in the upper leaflet retrieved via the MDAnalysis LeafletFinder module, we first identified those with at least one atom within 0.4 nm (using the capped_distance function in MDAnalysis 2.0)[29,30] from a minimum of 15 protein atoms, and we selected those that satisfy this criteria for 60% of the frames. For each selected ligand molecule, the list of protein residues satisfying the cutoff and criteria above was retrieved, and all the lists were compared, and merged together if 75% of the residues were identical. The percentage of identity was calculated by diving the total number of identical residues between two lists by the number of residues in the shorter list. The lists of protein residues obtained from this for LIN-S and LIN were then compared and merged together as belonging to the same region if sharing 80% of identity. The final regions identified by this procedure were then mapped on the protein and those corresponding to the location of sites 2 were selected. With this procedure we ensured that all residues interacting with ligands at these sites are taken into account,

and given the presence of both LIN-S and LIN at all four sites 2 in the systems, the hydrogen bond count described below captured some differences between LIN-S and LIN.

Hydrogen bond analysis. The total number of hydrogen bonds between any LIN-S or LIN molecule and (i) the selected protein residues for sites 2, (ii) the remaining protein residues interacting in the upper leaflet, and (iii) the protein residues of the lower leaflet was performed by means of the Hydrogen Bond Analysis module from MDAnalysis 2.0.[29,30] The last 1 μs of simulation time for each system, with frames saved every 1 ns, was used. For each residue, the total number of hydrogen bonds was scaled by the total number of frames in each trajectory.

Isolated heart experiments

Female Dunking Hartley guinea pigs (300-440 g) were obtained from Charles River, France and were 4 weeks old at the time of arrival. The animals were housed together in a pen with wood shavings as bedding and were provided with red polycarbonate enrichment tunnels and huts. They were kept at room temperature (21°C) at 12 hour light/dark cycle with *ad libitum* access to water and were fed daily. The guinea pigs were monitored daily by trained caretaker. A total of 16 adult female Dunkin Hartley guinea pigs were used for this study. The sample size was calculated based on previous studies,[32] expecting a standard deviation (SD) of the QT measurements of 11 ms and aiming for an effect size of Δ= 20 ms. The needed group sample size for the experiment was calculated using the formula:

$$n = \frac{2\ (Z_{\alpha} +\ Z_{1-\beta})^{2\sigma 2,}}{\Delta^2}$$

Where n is the required sample size. Z_{α} and $Z_{1-\beta}$are constants reflecting the alfa error (5%) of a 2-sided experiment and beta the power (80%) of the study. The estimated sample size was n = 5.

No animals were excluded before surgery, but issues arisen during surgery leading to exclusion include cardiac arrest from anesthesia, inability to maintain a constant perfusion pressure during

stabilization period, loss of electrical signals during experiment, or hearts having an intrinsic heart rate above 250 BPM at the end of the 30 min stabilization period. Four experiments were excluded because of instrumentation issues or other irregularities. The study was not randomized or blinded. The guinea pigs were transported to the laboratory in type 3 cages with wood shaving bedding and fresh straw for cover. No adverse events were monitored. The guinea pigs were anaesthetized by an intraperitoneal injection of 1.5 mL/kg pentobarbital and lidocaine hydrochloride (200 mg/mL and 20 mg/mL) from Glostrup Apotek, Denmark. The depth of anesthesia was monitored by checking for reflexes when the paws were pinched. If the depth of anesthesia was not sufficient, the animal was left for additional 5 minutes until no reflexes were present. Following the animal experimental license, the human endpoints were euthanasia with intraperitoneal injection of pentobarbital if any animals were not thriving or were sick. Animals were monitored daily.

The guinea pigs were artificially ventilated via the trachea using a rodent ventilator (Ugo Basile Model 7025) set with 60 strokes/min and 5 mL volume. The hearts were excised through a thoracotomy, cannulated in-situ, and connected to the Langendorff apparatus (Hugo Sachs, Harvard Apparatus). Once cannulated, the hearts were perfused under a constant pressure of 60 mmHg and submerged in 37°C Krebs-Henseleit buffer. The buffer contained (mmol/L): 120 NaCl, 25 $NaHCO_3$, 4 KCl, 0.6 $MgSO_4$, 0.6 NaH_2PO_4, 2.5 $CaCl_2$, 11 glucose and was continuously gassed with a mixture of 95% oxygen (O_2) and 5% carbon dioxide (CO_2) via a sintered glass gas diffuser. The Langendorff system was connected to a signal amplifier (Hugo Sachs Elektronik-Harvard Apparatus GmbH, Germany) and data were continuously sampled at 2 KHz using the 16-channel PowerLab system (ADInstruments, Oxford, UK), and monitoried by LabChart 8 software (ADInstruments). Volume conducted ECGs and three epicardial monophasic action potential (MAP) electrodes (Hugo Sachs Elektronik-Harvard Apparatus GmbH, March-Hugstetten, Germany) were positioned on the right atrial appendage and on both ventricles. A pacing electrode was placed on the right atrial appendage

and the hearts were paced at 240 basic cycle lengths/ 250 beats per minute with square pulses of 2 milliseconds at 2 times threshold.

After a 30-minute stabilization period, pacing was initiated for 2 minutes to obtain baseline recordings and followed by 20 minutes perfusion with 0.03 µM E4031. We have previously shown that this concentration prolongs the APD and QT interval[32,33] and is believed to exclusively affect the I_{Kr} current.[34,35] At the end of the 20 minutes perfusion period, the hearts were paced for 2 minutes at 250 BPM. This was followed by three 20 minutes perfusion periods with 0.03 µM E4031 and 1 µM, 3 µM and 10 µM ARA-S, or equivalent ethanol for time matched control. At the end of each perfusion period, the hearts were paced again for 2 minutes at 250 BPM for collection of electrophysiological parameters. QT interval, left and right ventricle APD_{90} were analysed using 50 consecutive beats at the end of the pacing period using LabChart 8.

Statistical analysis

Average values are expressed as mean ± SEM. Statistics were calculated using one-sample *t* test (to compare with a hypothetical value of 0), Student´s *t* test to compare between two groups or one-way ANOVA followed by Tukey´s multiple comparisons test to compare multiple groups. For the isolated heart experiments, two-way ANOVA with Dunnett´s multiple comparisons test was used. $P < 0.05$ was considered statistically significant. The QTc data was normally distributed according to a Shapiro-Wilk normality test. All statistical analyses were carried out in GraphPad Prism 9.

Role of the funding source

The funding sources had not involvement in the study design, in the collection, analysis, and interpretation of data, or in the writing of the manuscript.

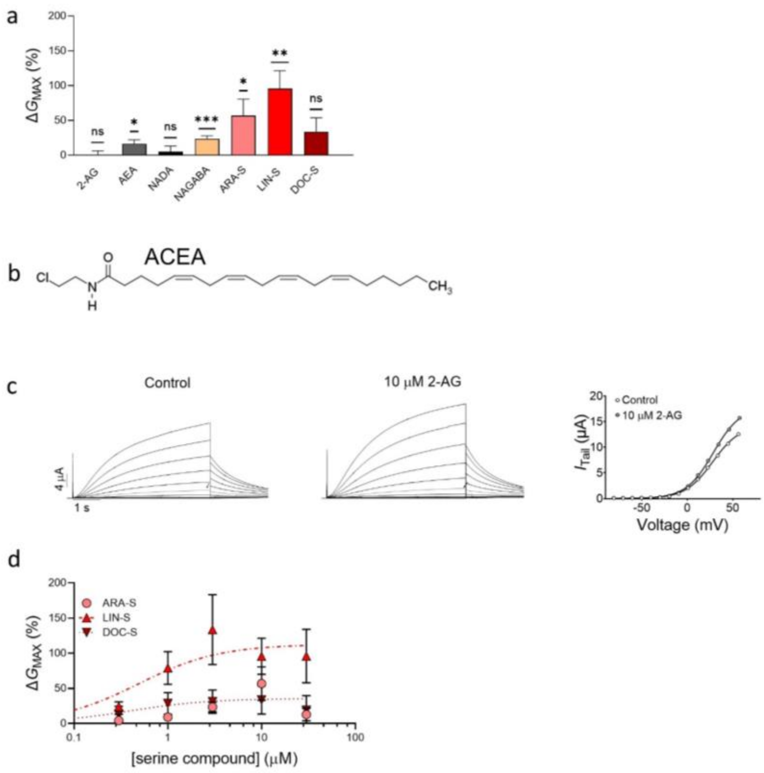

Supplementary figure 1. G_{MAX} effect of endocannabinoids. Effect of indicated endocannabinoids on $hK_V7.1$/KCNE1 expressed in *Xenopus* oocytes and studied with the two-electrode voltage clamp technique. a) ΔG_{MAX} induced by 10 µM of indicated endocannabinoids on $hK_V7.1$/KCNE1. Statistics indicate one-sample *t* test compared to a hypothetical value of 0 (i.e. no change in G_{MAX}). * denotes $P < 0.05$, ** denotes $P < 0.01$, *** denotes $P < 0.001$. $P > 0.05$ (ns). b) Structure of ACEA. c) Representative traces of $hK_V7.1$/KCNE1 currents under control conditions and in the presence of 10 µM 2-AG (grey traces indicate an activating voltage step to 0 mV) and corresponding G(V) curves. Curves in the G(V) plot (right) represent Boltzmann fits. For this specific cell: $V_{50;ctrl}$ = +27.9 mV, $I_{tailmax;ctrl}$ = 14.4 µA, $V_{50;2\text{-}AG}$ = +28.5 mV, $I_{tailmax;2\text{-}AG}$ = 17.9 µA. d) Concentration-response relation for ΔG_{MAX} effect of ARA-S, LIN-S and DOC-S. Best fit = ambiguous. Data shown as mean ± SEM; n = 7-12. Note that the 30 µM concentration was excluded from the fit.

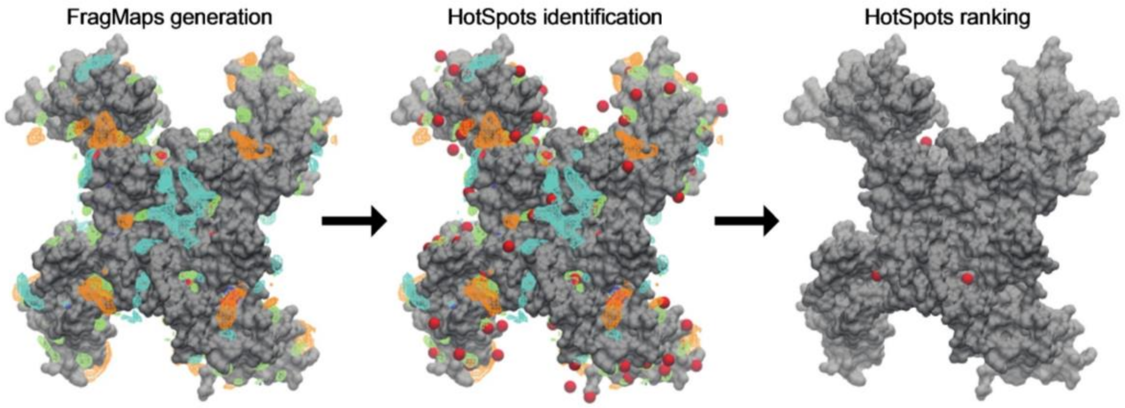

Supplementary figure 2. SILCS workflow. Overview of the SILCS workflow to identify putative interaction regions of a ligand. For a given protein, SILCS protocol includes the generation of FragMaps (left panel), i.e. free energy maps for different functional groups, then used to identify HotSpots (middle panel), putative regions of interaction for the ligands of choice. The HotSpots are then ranked (right panel) based on the Ligand Grid Free Energy score and those with the best LGFE values are used for further analysis. Shown are FragMaps obtained from *Xenopus laevis* $K_V7.1$, represented as mesh at different GFE cut-off levels: apolar (green, −0.9 kcal/mol), hydrogen bond acceptor (red, −0.6 kcal/mol), hydrogen bond donor (blue, −0.6 kcal/mol), negatively (orange −1.2 kcal/mol) and positively charged (cyan, −1.2 kcal/mol) functional groups.

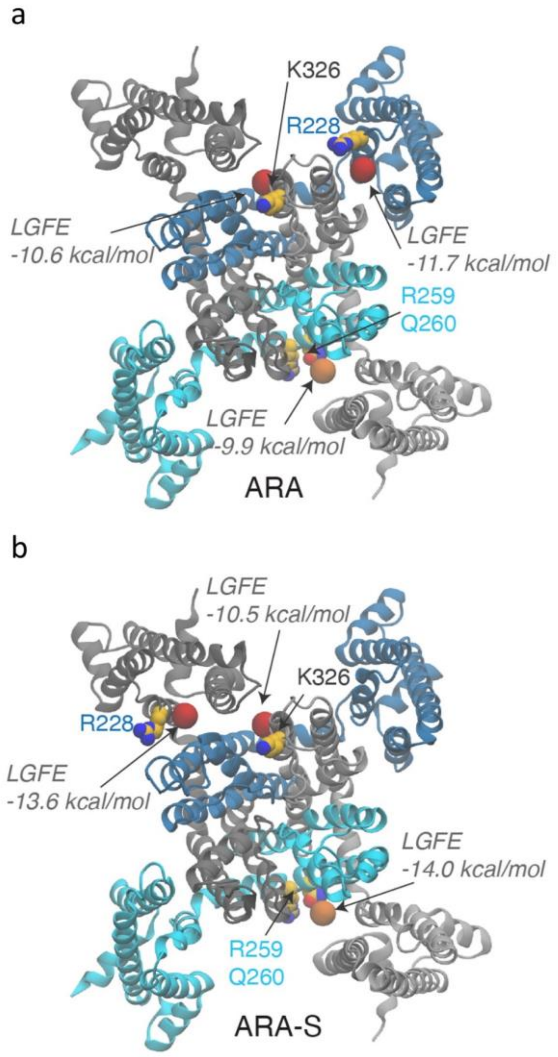

Supplementary figure 3. SILCS regions of interactions of ARA and ARA-S with K_V7.1. Outcome of computational analysis of ARA and ARA-S interaction with K_V7.1. The centroids of the identified regions of interactions for a) ARA and b) ARA-S are shown as red and orange spheres for regions in the upper and lower leaflet, respectively. The SILCS score for each region is reported, and known PUFAs binding residues at sites 1 (R228) and 2 (K326)[36] and additional residues in the lower leaflet are shown as spheres, as a reference.

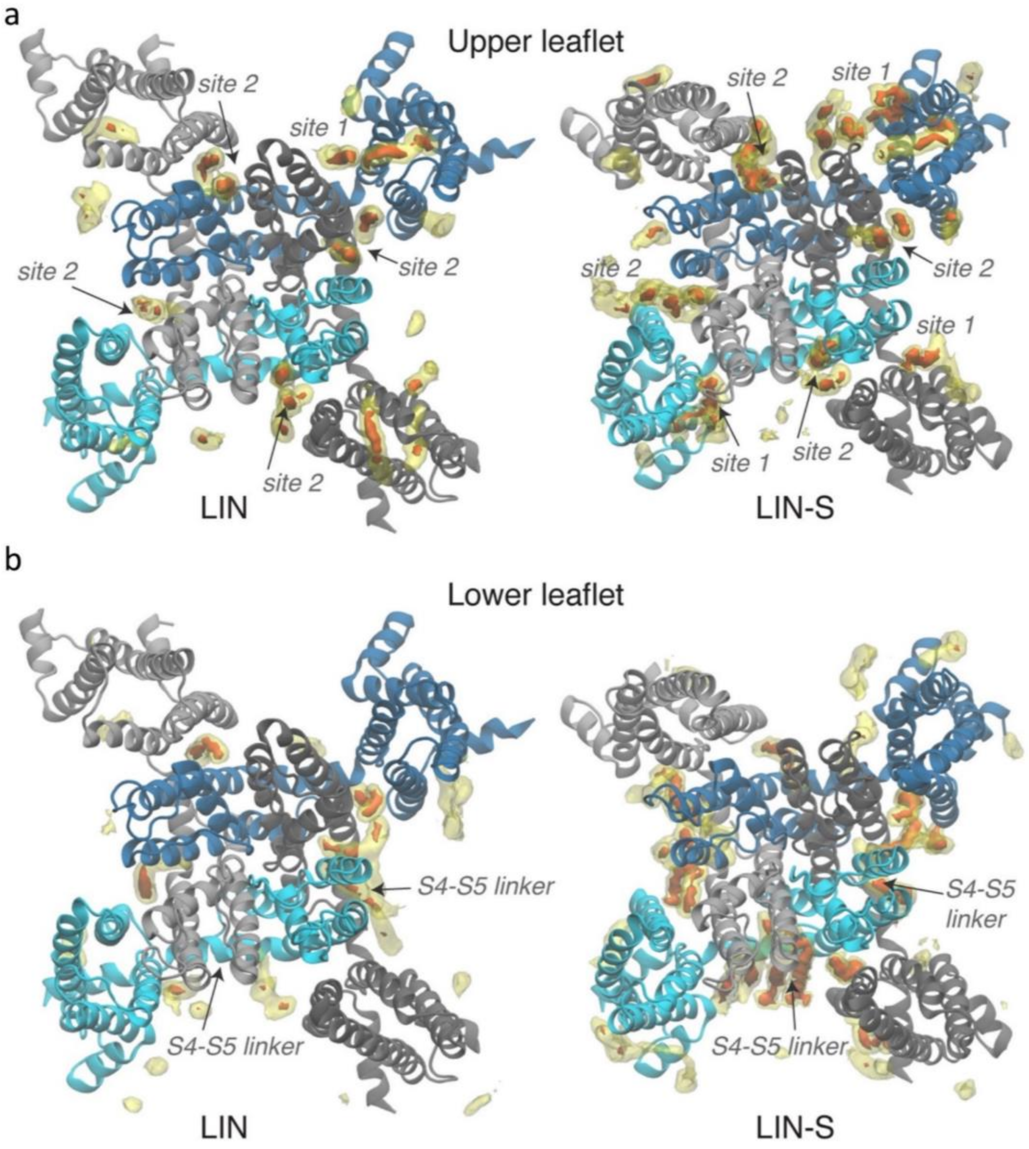

Supplementary figure 4. LIN and LIN-S density around K_V7.1. Outcome of computational analysis of LIN and LIN-S interaction with hK_V7.1. The number density was obtained from the last 1 µs of simulation time for the LIN (left panels) and LIN-S (right panels) systems, and it is shown as isosurfaces drawn at 0.19 (in red) and 0.09 (in yellow) for a) the upper and b) the lower leaflet. In all panels, the protein represents the last frame of each simulation system. Known binding sites for PUFAs (namely site 1 and site 2)[36] and the linker connecting the S4 and S5 segments of one monomer are highlighted as a reference. In all panels, the protein is viewed from the extracellular side.

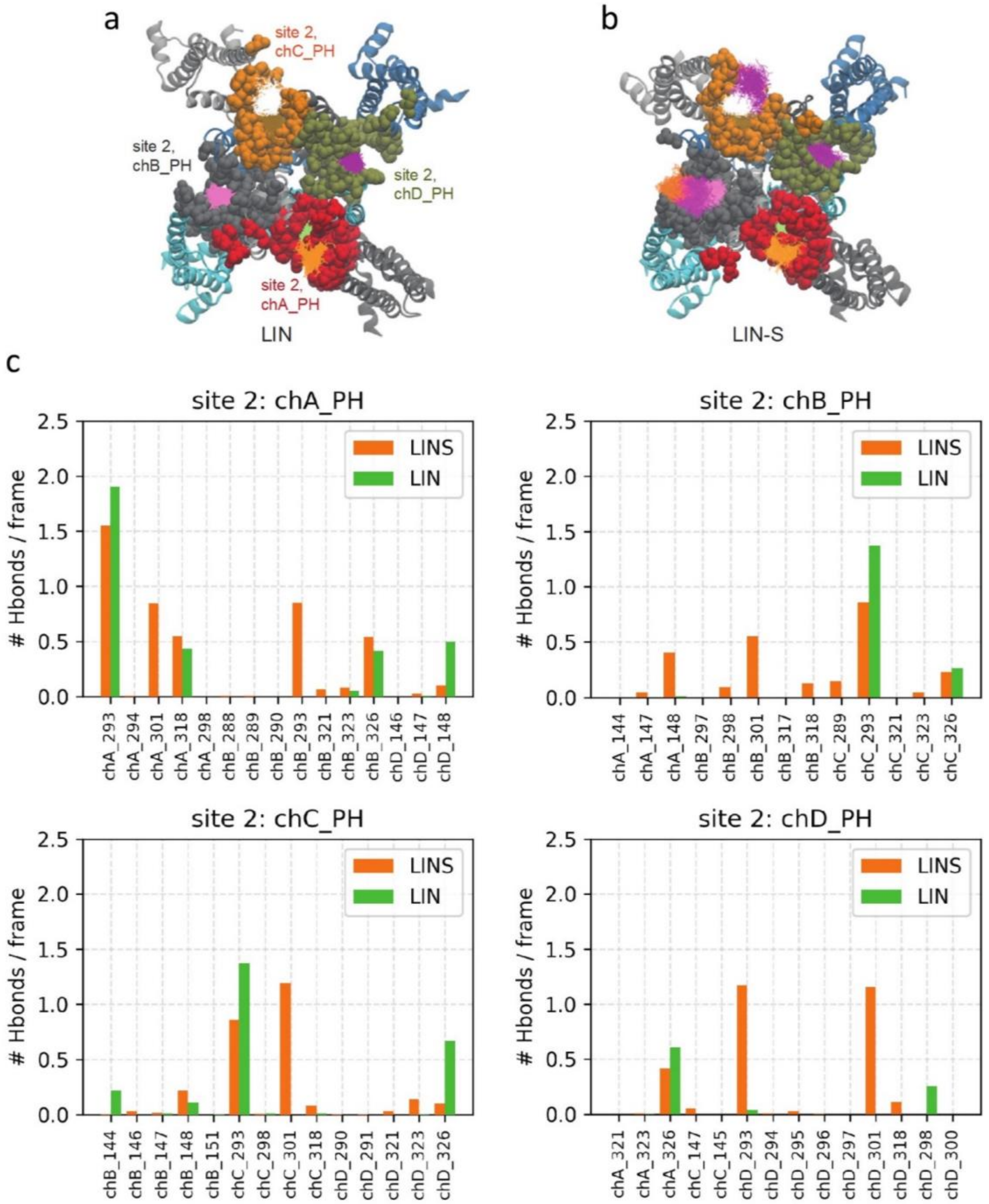

Supplementary figure 5. LIN and LIN-S at sites 2 and hydrogen bonds count. Outcome of computational analysis of LIN and LIN-S interaction with hK$_V$7.1, with focus on site 2. The residues of the four common regions occupied by a) LIN and b) LIN-S corresponding to sites 2 are shown as spheres, colored in red, gray, orange and olive green. The sites are mapped onto the last frame of the simulation systems. Examples of ligand molecules found at each region are depicted as lines,

with frames taken every 2 ns during the last 1 µs of the simulations. c) For each site, the bar graphs show the total number of hydrogen bonds detected between the protein residues of the selected site and the two ligands, normalized by the total number of frames used for the analysis.

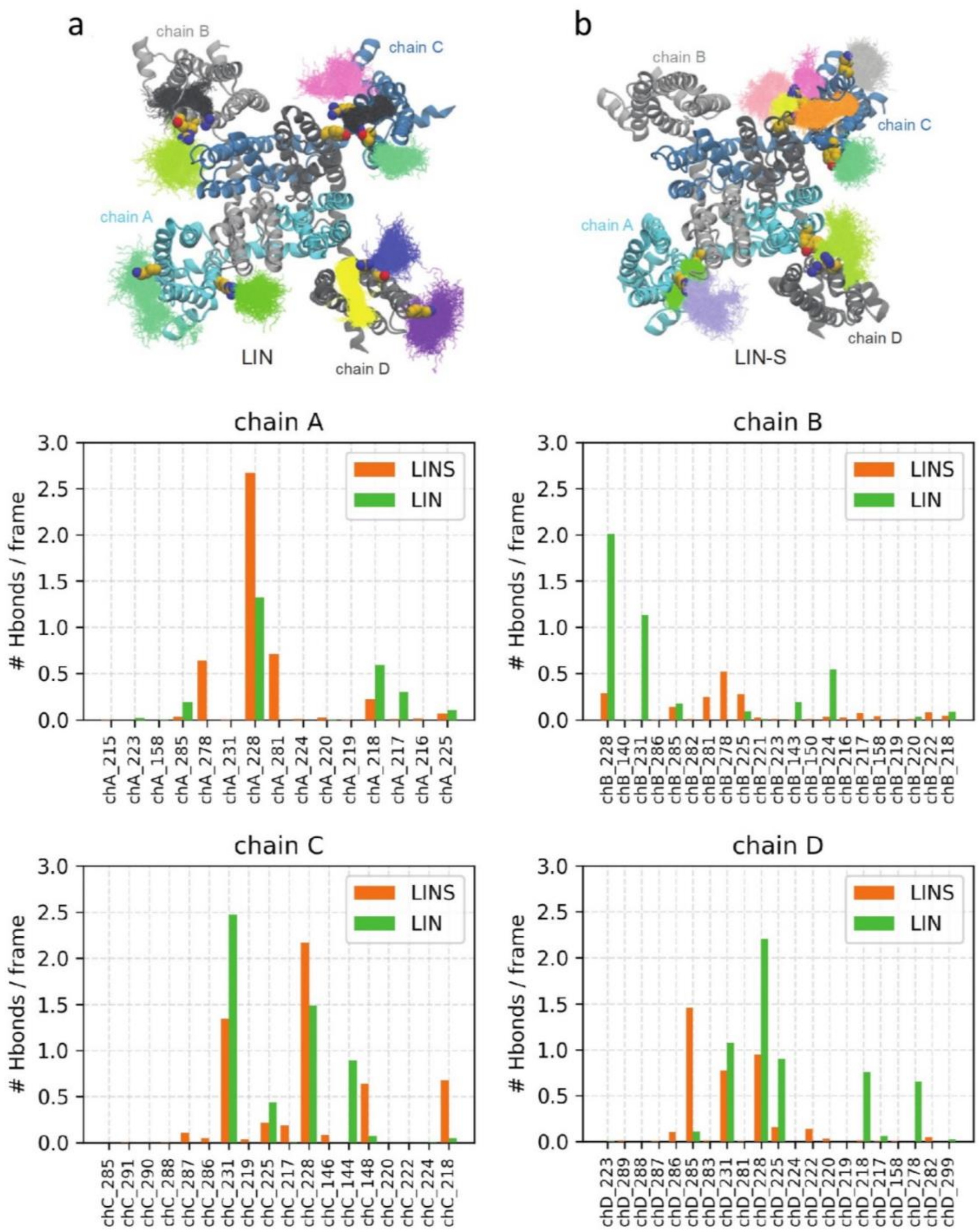

Supplementary figure 6. LIN and LIN-S at additional interaction regions in the upper leaflet and hydrogen bond count. Outcome of computational analysis of LIN and LIN-S interaction with $hK_V7.1$, with focus on site 1 and additional regions in the upper leaflet. Examples of a) LIN and b) LIN-S molecules found at sites 1 or at other VSD regions in the upper leaflet are depicted as lines, with frames taken every 2 ns during the last 1 µs of the simulations. The protein corresponds to the last frame of the simulations. c) For each site, the bar graphs show the total number of

hydrogen bonds detected between the protein residues of the selected site and the two ligands, normalized by the total number of frames used for the analysis.

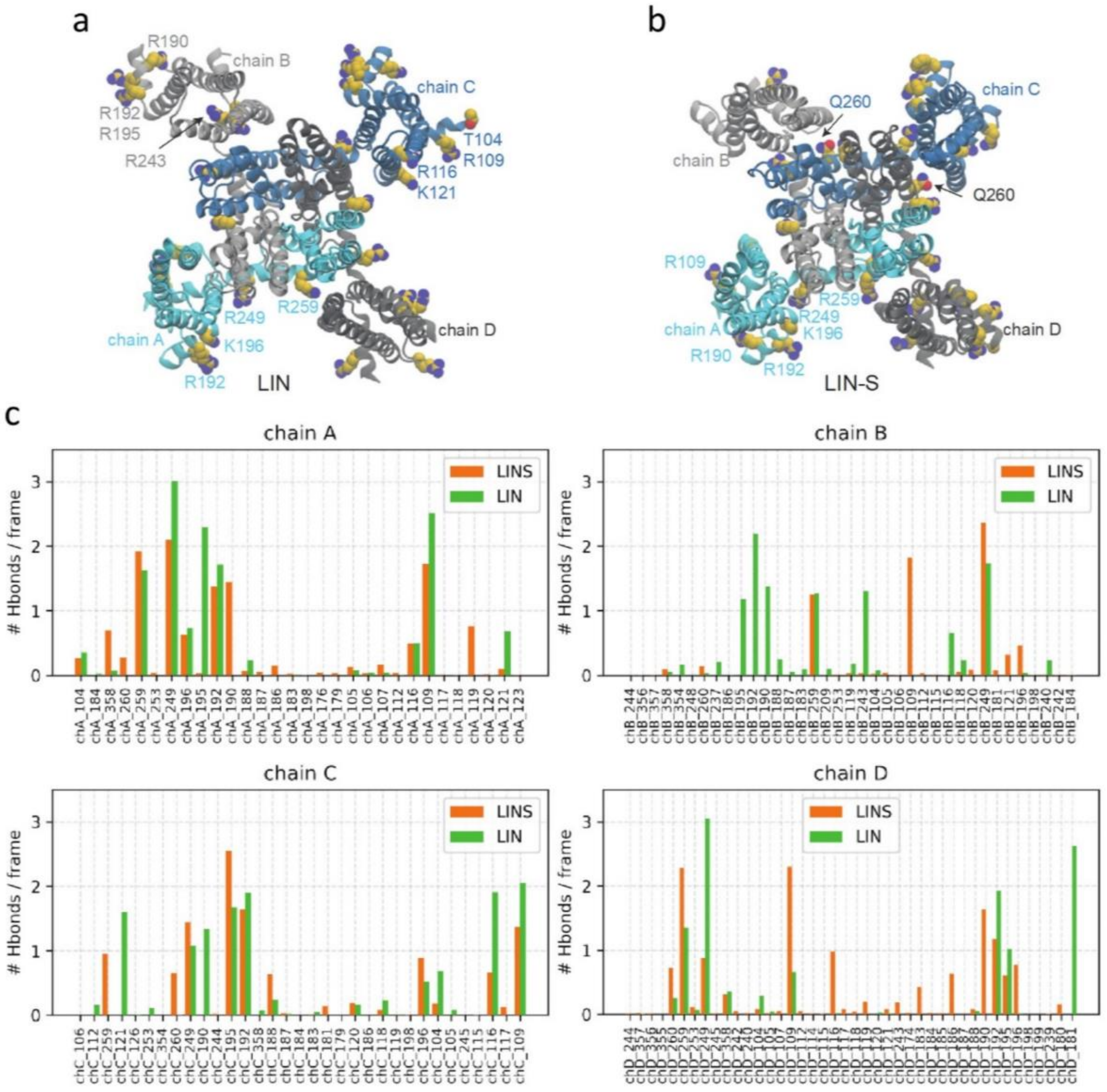

Supplementary figure 7. Residues engaging in hydrogen bonds with LIN and LIN-S in the lower leaflet. Outcome of computational analysis of LIN and LIN-S interaction with $hK_V7.1$, with focus on the inner leaflet. a-b) Protein residues engaged in the highest number of hydrogen bonds with (a) LIN and (b) LIN-S in the lower leaflet. Residues are labelled according to the color of the corresponding monomer, cyan for chain A, light gray for chain B, blue for chain C and gray for chain D. For clarity, not all residues were labelled. The total count of the hydrogen bonds was scaled by the number of frames, and only residues forming a minimum of 0.5 hydrogen bonds per

frame are shown. In all panels, the protein represents the last frame of each simulation system. c) For each monomer residue, the bar graphs show the total number of hydrogen bonds detected between the protein residues of the selected site and the two ligands, normalized by the total number of frames used for the analysis.

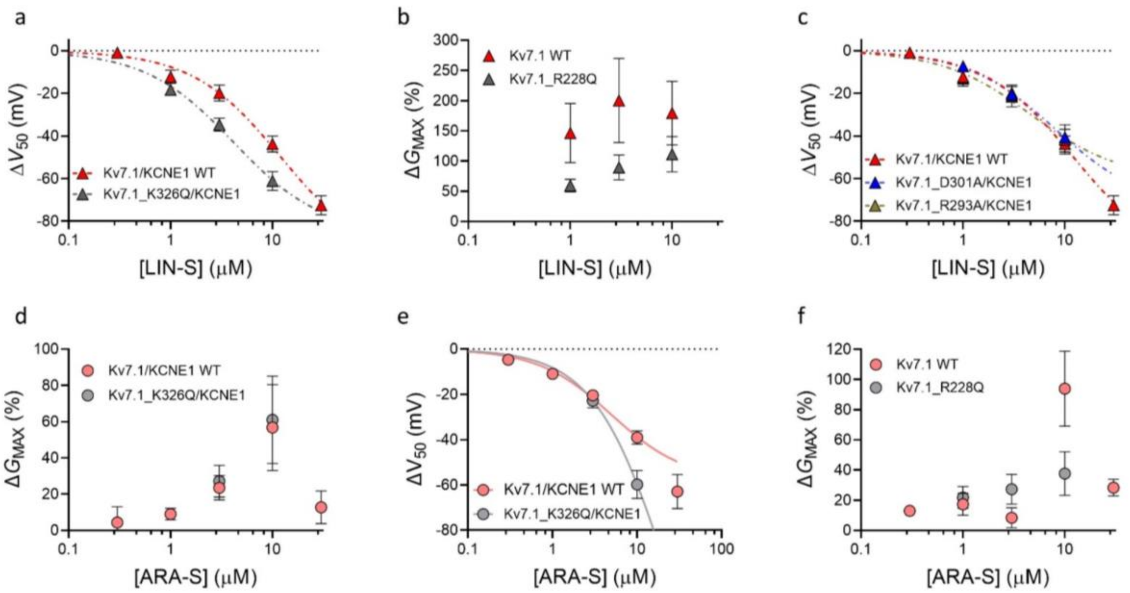

Supplementary figure 8. Maintained effects of LIN-S on Site 2 mutations, G_{MAX} of hK_V7.1_R228Q and ARA-S G_{MAX} effect on hK_V7.1_K326Q/KCNE1. Effect of LIN-S or ARA-S on indicated hK_V7.1 or hK_V7.1/KCNE1 mutants expressed in *Xenopus* oocytes and studied with the two-electrode voltage clamp technique. a) Concentration-response relation for ΔV_{50} effect of LIN-S in hK_V7.1/KCNE1 WT and hK_V7.1_K326Q/KCNE1. Best fit for LIN-S in hK_V7.1_K326Q/KCNE1: EC_{50} = 4.1 µM, $\Delta V_{50,\ max}$ = -85.8 mV. Data shown as mean ± SEM; n = 4-10. b) Concentration-response relation for ΔG_{MAX} effect of LIN-S in hK_V7.1 WT and hK_V7.1_R228Q. Data shown as mean ± SEM; n = 4-6. c) Concentration-response relation for ΔV_{50} effect of LIN-S in hK_V7.1/KCNE1 WT, hK_V7.1_R293A/KCNE1 and hK_V7.1_D301A/KCNE1. Best fit for LIN-S in hK_V7.1_R293A/KCNE1: EC_{50} = 4.6 µM, $\Delta V_{50,\ max}$ = -60.1 mV. Best fit for LIN-S in hK_V7.1_D301A/KCNE1: EC_{50} = 8 µM, $\Delta V_{50,\ max}$ = -73.8 mV. Data shown as mean ± SEM; n = 4-10. d) Concentration-response relation for ΔG_{MAX} effect of ARA-S in hK_V7.1/KCNE1 WT and hK_V7.1_K326Q/KCNE1. Data shown as mean ± SEM; n = 4-12. e) Concentration-response relation for ΔV_{50} effect of ARA-S in hK_V7.1/KCNE1 WT and hK_V7.1_K326Q/KCNE1. Data shown as mean ± SEM; n = 4-12. f) Concentration-response relation for ΔG_{MAX} effect of ARA-S in hK_V7.1 WT and hK_V7.1_R228Q. Data shown as mean ± SEM; n = 4-9. No fit in b, d, and f were made because of the lack of robust G_{MAX} effects at the highest concentration.

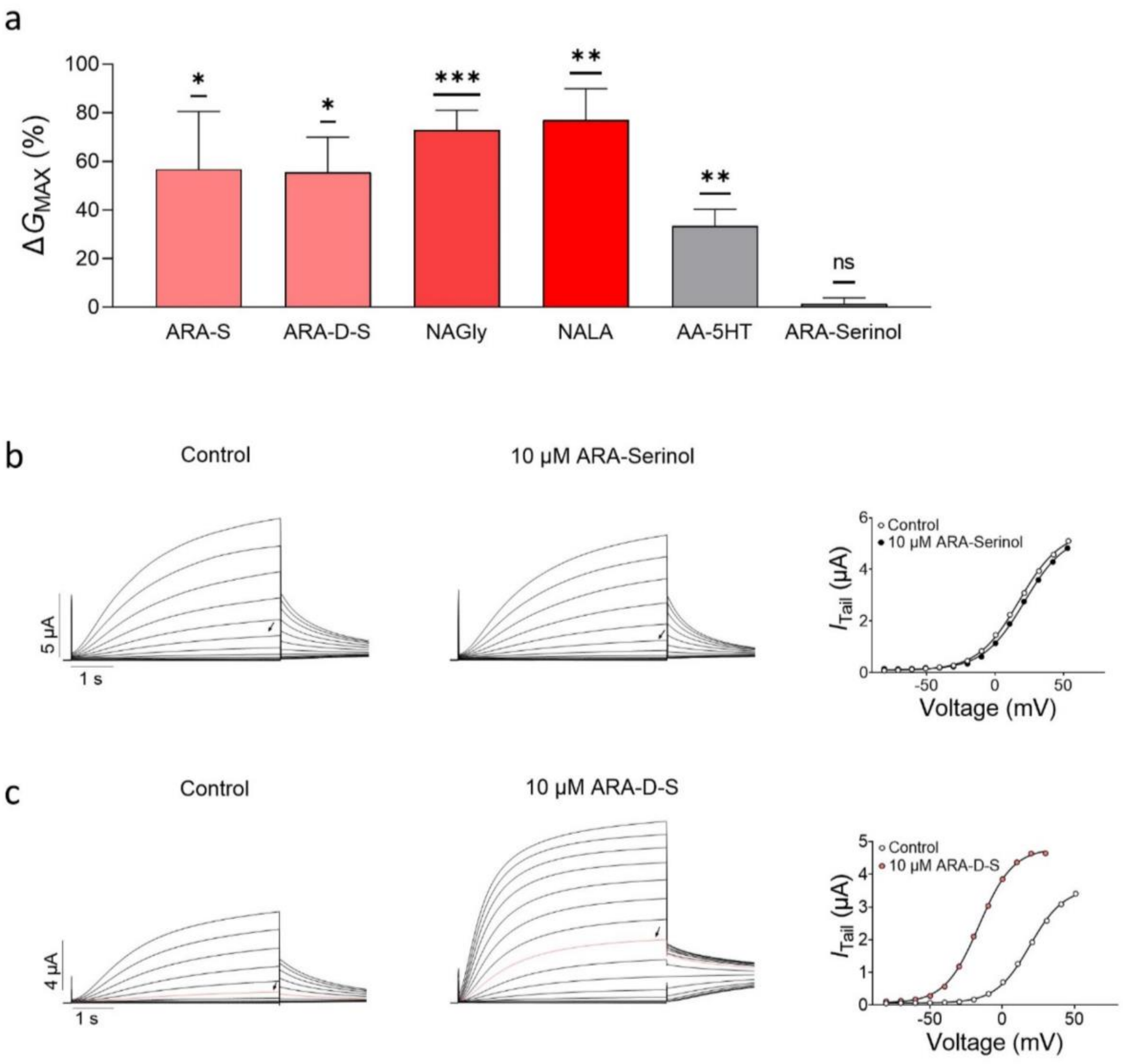

Supplementary Figure 9. Effect of endocannabinoid head group properties and KCNE1 for the ARA-S effect. Effect of indicated arachidonic acid-based compounds on $hK_V7.1$/KCNE1 expressed in *Xenopus* oocytes and studied with the two-electrode voltage clamp technique. a) Mean ΔG_{MAX} induced by 10 µM of ARA-S, ARA-D-S, NAGly, NALA, AA-5HT and ARA-Serinol on $hK_V7.1$/KCNE1. Statistics indicate one-sample *t* test compared to a hypothetical value of 0 (i.e. no change in V_{50} or current amplitude). * denotes P < 0.05, ** denotes P < 0.01, *** denotes P < 0.001. P > 0.05 (ns). Data shown as mean ± SEM. n = 6-12. b) Representative traces of $hK_V7.1$/KCNE1 currents under control conditions and in the presence of 10 µM ARA-Serinol and corresponding G(V) curves. Curves in the G(V) plot (right) represent

Boltzmann fits. For this specific cell: $V_{50;ctrl}$ = +17.6 mV, $I_{tailmax;ctrl}$ = 5.5 µA, $V_{50;ARA\text{-}Serinol}$ = +21.4 mV, $I_{tailmax;ARA\text{-}Serinol}$ = 5.4 µA. c) Representative traces of hK_V7.1/KCNE1 currents under control conditions and in the presence of 10 µM ARA-D-S and corresponding G(V) curves. Curves in the G(V) plot (right) represent Boltzmann fits. For this specific cell: $V_{50;ctrl}$ = +19.1 mV, $I_{tailmax;ctrl}$ = 3.6 µA, $V_{50;ARA\text{-}D\text{-}S}$ = -16.8 mV, $I_{tailmax;ARA\text{-}D\text{-}S}$ = 4.7 µA.

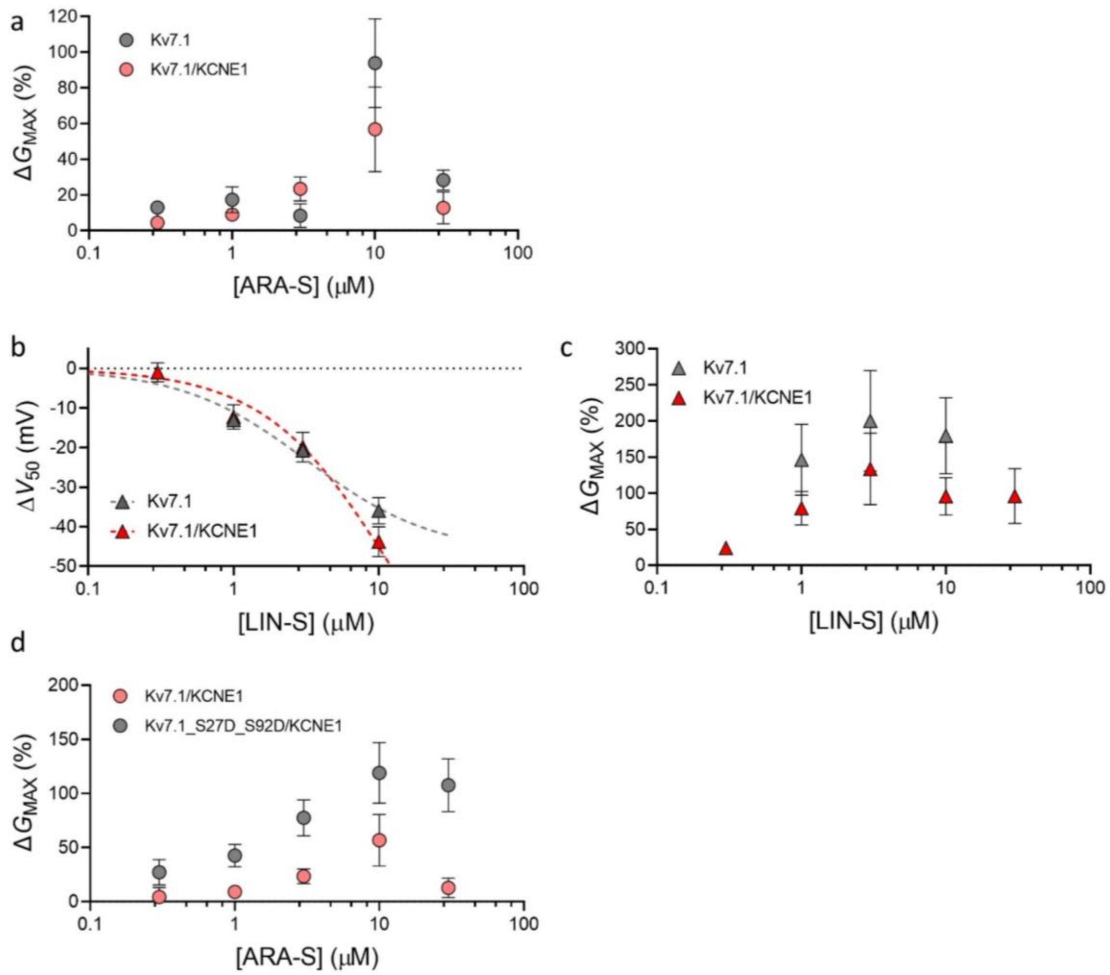

Supplementary figure 10. Effect of KCNE1 and phosphomimetic mutations for the endocannabinoid effect. Effect of LIN-S or ARA-S on $hK_V7.1$ or $hK_V7.1$/KCNE1, or indicated mutant, expressed in *Xenopus* oocytes and studied with the two-electrode voltage clamp technique. a) ΔG_{MAX} of ARA-S in $K_V7.1$ with and without KCNE1. Data shown as mean ± SEM; n = 4-12. The effect of 10 µM of ARA-S is comparable to the effect previously reported[1]. b) Concentration-response relation for the V_{50} effect of LIN-S on $hK_V7.1$ with and without KCNE1. Best fit for $hK_V7.1$: EC_{50} = 3.2 µM, $\Delta V_{50,\ max}$ = -46.7 mV. Data shown as mean ± SEM; n = 4-10. c) Same as in B but for ΔG_{MAX}. Data shown as mean ± SEM; n = 4-10. d) Concentration-response relation for ΔG_{MAX} of ARA-S in $hK_V7.1$/KCNE1 and $hK_V7.1$_S27D_S92D/KCNE1. Data shown as mean ± SEM; n = 5-13. No fit in a, c, and d were made because of the lack of robust G_{MAX} effects at the highest concentration.

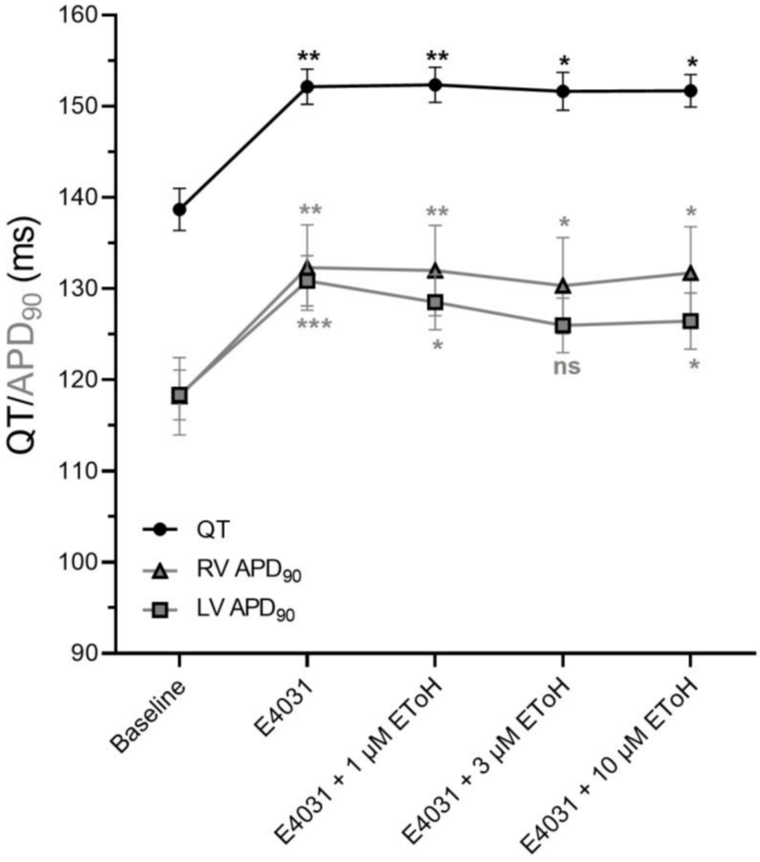

Supplementary figure 11. Time-matched control experiments for guinea heart experiments.

Time-matched control experiments of vehicle only in isolated guinea pig experiments. Summary of the changes in QT interval and action potential duration induced by E4031 alone or E4031 co-applied with vehicle. Statistics represent two-way ANOVA with Dunnett´s multiple comparisons test and indicates the difference compared to baseline. * denotes P < 0.05, ** denotes P < 0.01. P > 0.05 (ns). Data shown as mean ± SEM; n = 6.

[1] Larsson JE, Karlsson U, Wu X, Liin SI. Combining endocannabinoids with retigabine for enhanced M-channel effect and improved KV7 subtype selectivity. *J Gen Physiol* 2020; **152**(8).

[2]Redford KE, Rognant S, Jepps TA, Abbott GW. KCNQ5 Potassium Channel Activation Underlies Vasodilation by Tea. *Cell Physiol Biochem* 2021; **55**(S3): 46-64.

[3]O'Hara T, Virag L, Varro A, Rudy Y. Simulation of the undiseased human cardiac ventricular action potential: model formulation and experimental validation. *PLoS computational biology* 2011; **7**(5): e1002061.

[4]Piacentino V, 3rd, Weber CR, Chen X, Weisser-Thomas J, Margulies KB, Bers DM, et al. Cellular basis of abnormal calcium transients of failing human ventricular myocytes. *Circulation research* 2003; **92**(6): 651-8.

[5]Kruse M, Hammond GR, Hille B. Regulation of voltage-gated potassium channels by PI(4,5)P2. *J Gen Physiol* 2012; **140**(2): 189-205.

[6]Murata Y, Iwasaki H, Sasaki M, Inaba K, Okamura Y. Phosphoinositide phosphatase activity coupled to an intrinsic voltage sensor. *Nature* 2005; **435**(7046): 1239-43.

[7]Loussouarn G, Park KH, Bellocq C, Baró I, Charpentier F, Escande D. Phosphatidylinositol-4,5-bisphosphate, PIP2, controls KCNQ1/KCNE1 voltage-gated potassium channels: a functional homology between voltage-gated and inward rectifier K+ channels. *EMBO J* 2003; **22**(20): 5412-21.

[8]Guvench O, MacKerell AD, Jr. Computational fragment-based binding site identification by ligand competitive saturation. *PLoS Comput Biol* 2009; **5**(7): e1000435.

[9]MacKerell AD, Jo S, Lakkaraju SK, Lind C, Yu W. Identification and characterization of fragment binding sites for allosteric ligand design using the site identification by ligand competitive saturation hotspots approach (SILCS-Hotspots). *Biochimica et Biophysica Acta (BBA) - General Subjects* 2020; **1864**(4): 129519.

[10]Raman EP, Yu W, Lakkaraju SK, MacKerell AD. Inclusion of Multiple Fragment Types in the Site Identification by Ligand Competitive Saturation (SILCS) Approach. *Journal of Chemical Information and Modeling* 2013; **53**(12): 3384-98.

[11]Ustach VD, Lakkaraju SK, Jo S, Yu W, Jiang W, MacKerell AD. Optimization and Evaluation of Site-Identification by Ligand Competitive Saturation (SILCS) as a Tool for Target-Based Ligand Optimization. *Journal of Chemical Information and Modeling* 2019; **59**(6): 3018-35.
[12]Sun J, MacKinnon R. Cryo-EM Structure of a KCNQ1/CaM Complex Reveals Insights into Congenital Long QT Syndrome. *Cell* 2017; **169**(6): 1042-50 e9.
[13]Jo S, Kim T, Iyer VG, Im W. CHARMM-GUI: a web-based graphical user interface for CHARMM. *J Comput Chem* 2008; **29**(11): 1859-65.
[14]Klauda JB, Venable RM, Freites JA, O'Connor JW, Tobias DJ, Mondragon-Ramirez C, et al. Update of the CHARMM all-atom additive force field for lipids: validation on six lipid types. *J Phys Chem B* 2010; **114**(23): 7830-43.
[15]Huang J, Rauscher S, Nawrocki G, Ran T, Feig M, de Groot BL, et al. CHARMM36m: an improved force field for folded and intrinsically disordered proteins. *Nat Methods* 2017; **14**(1): 71-3.
[16]Jorgensen WL, Chandrasekhar J, Madura JD, Impey RW, Klein ML. Comparison of simple potential functions for simulating liquid water. *The Journal of Chemical Physics* 1983; **79**(2): 926-35.
[17]Vanommeslaeghe K, Hatcher E, Acharya C, Kundu S, Zhong S, Shim J, et al. CHARMM general force field: A force field for drug-like molecules compatible with the CHARMM all-atom additive biological force fields. *J Comput Chem* 2010; **31**(4): 671-90.
[18]Vanommeslaeghe K, MacKerell AD, Jr. Automation of the CHARMM General Force Field (CGenFF) I: bond perception and atom typing. *J Chem Inf Model* 2012; **52**(12): 3144-54.
[19]Hoover WG. Canonical dynamics: Equilibrium phase-space distributions. *Physical Review A* 1985; **31**(3): 1695-7.
[20]Nosé S. A molecular dynamics method for simulations in the canonical ensemble. *Molecular Physics* 1984; **52**(2): 255-68.
[21]Parrinello M, Rahman A. Polymorphic transitions in single crystals: A new molecular dynamics method. *Journal of Applied Physics* 1981; **52**(12): 7182-90.

[22]Foster TJ, MacKerell AD, Jr., Guvench O. Balancing target flexibility and target denaturation in computational fragment-based inhibitor discovery. *Journal of computational chemistry* 2012; **33**(23): 1880-91.

[23]Sun J, MacKinnon R. Structural Basis of Human KCNQ1 Modulation and Gating. *Cell* 2020; **180**(2): 340-7.e9.

[24]Noskov SY, Im W, Roux B. Ion permeation through the alpha-hemolysin channel: theoretical studies based on Brownian dynamics and Poisson-Nernst-Plank electrodiffusion theory. *Biophys J* 2004; **87**(4): 2299-309.

[25]Noskov SY, Roux B. Control of ion selectivity in LeuT: two Na+ binding sites with two different mechanisms. *J Mol Biol* 2008; **377**(3): 804-18.

[26]Pronk S, Páll S, Schulz R, Larsson P, Bjelkmar P, Apostolov R, et al. GROMACS 4.5: a high-throughput and highly parallel open source molecular simulation toolkit. *Bioinformatics* 2013; **29**(7): 845-54.

[27]Hess B, Bekker H, Berendsen HJC, Fraaije JGEM. LINCS: A linear constraint solver for molecular simulations. *Journal of Computational Chemistry* 1997; **18**(12): 1463-72.

[28]Essmann U, Perera L, Berkowitz ML, Darden T, Lee H, Pedersen LG. A smooth particle mesh Ewald method. *The Journal of Chemical Physics* 1995; **103**(19): 8577-93.

[29]Michaud-Agrawal N, Denning EJ, Woolf TB, Beckstein O. MDAnalysis: a toolkit for the analysis of molecular dynamics simulations. *J Comput Chem* 2011; **32**(10): 2319-27.

[30]Gowers R, Linke M, Barnoud J, Reddy T, Melo M, Seyler S, et al. MDAnalysis: A Python Package for the Rapid Analysis of Molecular Dynamics Simulations; 2016.

[31]Humphrey W, Dalke A, Schulten K. VMD: visual molecular dynamics. *J Mol Graph* 1996; **14**(1): 33-8, 27-8.

[32]Skarsfeldt MA, Liin SI, Larsson HP, Bentzen BH. Polyunsaturated fatty acid-derived IKs channel activators shorten the QT interval ex-vivo and in-vivo. *Acta Physiol (Oxf)* 2020; **229**(4): e13471.

[33]Liin SI, Silverå Ejneby M, Barro-Soria R, Skarsfeldt MA, Larsson JE, Starck Härlin F, et al. Polyunsaturated fatty acid analogs act antiarrhythmically on the cardiac IKs channel. *Proc Natl Acad Sci U S A* 2015; **112**(18): 5714-9.

[34]Wettwer E, Scholtysik G, Schaad A, Himmel H, Ravens U. Effects of the new class III antiarrhythmic drug E-4031 on myocardial contractility and electrophysiological parameters. *J Cardiovasc Pharmacol* 1991; **17**(3): 480-7.

[35]Kügler P, Rast G, Guth BD. Comparison of in vitro and computational experiments on the relation of inter-beat interval and duration of repolarization in a specific type of human induced pluripotent stem cell-derived cardiomyocytes. *PLoS One* 2019; **14**(9): e0221763.

[36]Yazdi S, Nikesjö J, Miranda W, Corradi V, Tieleman DP, Noskov SY, et al. Identification of PUFA interaction sites on the cardiac potassium channel KCNQ1. *J Gen Physiol* 2021; **153**(6).
